# Intestinal obstruction impairs feeding and promotes sleep in *Drosophila melanogaster*

**DOI:** 10.1126/sciadv.ady2183

**Published:** 2026-05-20

**Authors:** Cindy Reinger, Laura Blackie, Alexandra M. Medeiros, Hugo Gillet, Carolin Kring, Pedro Gaspar, Dafni Hadjieconomou, Michèle Sickmann, Markus Affolter, Irene Miguel-Aliaga, Martin Müller, Anissa Kempf

**Affiliations:** ^1^ Biozentrum, University of Basel, Spitalstrasse 41, 4056 Basel, Switzerland.; ^2^ Institute of Clinical Sciences, Faculty of Medicine, Imperial College London, Du Cane Road, London W12 0NN, UK.; ^3^ MRC Laboratory of Medical Sciences, Hammersmith Hospital Campus, Du Cane Road, London W12 0NN, UK.

## Abstract

At the onset of life, feeding must be initiated while developmental waste products—the meconium—need to be eliminated. Although these two fundamental physiological processes are interconnected, their mechanistic coupling remains poorly understood. Using *Drosophila* as a model system, we investigated the coordination of these processes. We show that meconium excretion starts shortly after eclosion and that feeding initiation begins only after partial meconium elimination. We identified a cis-regulatory element associated with the *apterous* gene that is required for proper hindgut development. Its disruption prevents meconium excretion and leads to intestinal obstruction. As a result, flies with a defective element avoid food and exhibit increased proboscis extension sleep. Experimental inhibition of excretion in freshly eclosed flies recapitulates these phenotypes, indicating that intestinal blockage is sufficient to impair feeding and alter sleep/wake states. The progression of phenotypes parallels hallmarks of mechanical gut obstruction in humans, suggesting that the observed effects may arise from broader physiological consequences of intestinal blockage. Our findings uncover a link between intestinal clearance, feeding, sleep, and survival, with potential implications for understanding similar processes across species.

## Teaser

Intestinal obstruction disrupts feeding and promotes sleep in freshly eclosed flies.

## INTRODUCTION

The survival of animals hinges on a precisely orchestrated interplay of physiological processes. Among these, meconium excretion and feeding initiation represent crucial developmental milestones that establish the foundation for independent nutrient acquisition and organismal growth across diverse taxa. Despite their apparent evolutionary conservation, the mechanisms coordinating these essential processes remain unexplored.

Meconium excretion marks a critical transition in early life. This process eliminates accumulated metabolic waste from embryonic or pupal development (in vertebrates and arthropods, respectively), manifested as an odorless, mucilaginous substance whose color varies from greenish-black to yellowish-red across species. In mammals, failure to expel this developmental byproduct has been linked to severe conditions such as cystic fibrosis (*1*, *2*), while its premature release can lead to meconium aspiration syndrome—a major cause of neonatal mortality (*3*).

Feeding initiation represents an equally pivotal milestone, triggering the first intake of external nutrients. This process activates metabolic pathways, stimulates gut motility, and enables somatic growth; yet, how the organism knows when to begin feeding remains unclear. Recent advances have illuminated the neurological underpinnings of feeding initiation in both vertebrates and arthropods (*4*–*10*), but potential connections to excretory processes remain unexplored.

Correlative evidence across diverse species hints at an intimate interdependence between meconium excretion and feeding initiation. Equine neonates with meconium retention show diminished feeding interest, myliobatiform stingrays eliminate meconium before initiating feeding, and human infants with meconium plug syndrome display difficulties in food intake (*11*–*13*). Could meconium excretion contribute to the timing of feeding initiation? Do these observations reflect a conserved developmental strategy across animal phylogeny? How might the precise timing of these processes influence organismal health and survival?

*Drosophila melanogaster* is a genetically tractable model organism widely used for studying fundamental physiological processes. Despite their evolutionary divergence, *Drosophila* and vertebrates share fundamental structural and functional characteristics in their digestive systems, making flies an excellent model for investigating universal principles of intestinal physiology (*14*–*17*). In this study, we investigate a century-old yet uncharacterized short-lived phenotype in *Drosophila* mutants lacking a functional *apterous* (*ap*) gene, best known for its role in wing development (*18*). By combining modern genetic tools with behavioral assays, we reveal unexpected links between intestinal blockage, hindgut function, sleep, and feeding.

## RESULTS

### Meconium excretion and adult feeding initiation are temporally coupled

Upon eclosion, several organs of newly eclosed flies undergo a series of developmental transitions to achieve full functional maturity, including the gut, the reproductive organs, the fat body, and the wings (*19*–*24*). The female reproductive organs, in particular the ovaries, remain underdeveloped at eclosion and require hormonal signals and nutrient availability to initiate vitellogenesis, which subsequently leads to a marked increase in ovary size (*25*, *26*). Similarly, the gut, initially filled with meconium, undergoes substantial remodeling and gradually adopts its adult structure over several days (*15*, *27*, *28*). This post-eclosion remodeling seems to be tightly coordinated with the initiation of adult feeding, ensuring a smooth transition from metamorphosis to adult life.

**Fig. 1.**
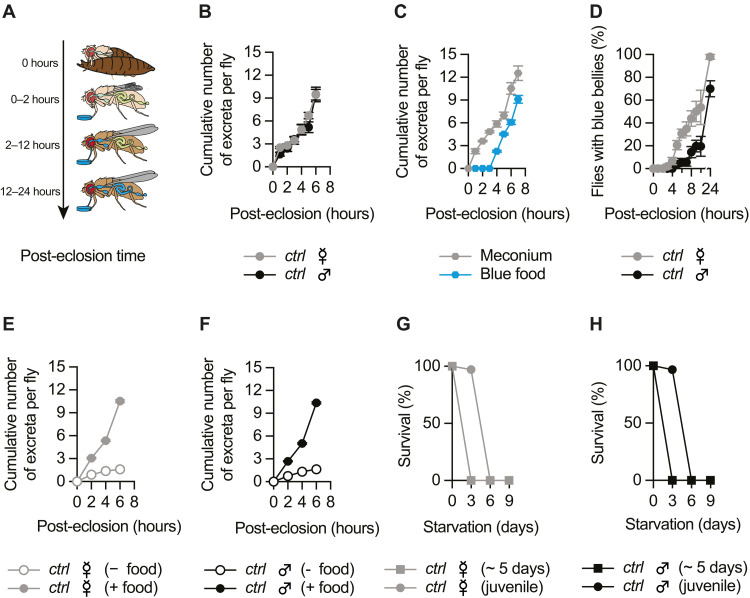
Meconium excretion is temporally coupled to feeding initiation. (**A**) Schematic overview of feeding-related physiological events during the first 24 hours post-eclosion in *D. melanogaster*. (**B**) Meconium excretion begins within the first hour after eclosion and occurs over a period of at least 6 hours in both sexes [time effect: *P* < 0.0001; time × gender interaction: *P* = 0.0792, two-way analysis of variance (ANOVA), *N* = 3]. (**C**) Time course of meconium and blue-dyed food excretion over the first 8 hours post-eclosion. The first digested food appears in excreta 4 hours post-eclosion (excreta type effect: *P* = 0.0001; excreta type × time interaction: *P* = 0.0078, Repeated Measures two-way ANOVA, *N* = 4). (**D**) Time course of blue-dyed food ingestion over the first 24 hours post-eclosion. The first *ctrl* flies initiate adult feeding ~3 hours post-eclosion. Females begin feeding earlier than males, and by 24 hours, all females have ingested food, whereas only 70% of males have done so (time × gender interaction: *P* = 0.0323, mixed-effects model, *N* = 4). (**E** and **F**) Meconium excretion rate is slower in the absence of food in female (E) and male (F) flies (time × food interaction: *P* < 0.0001, two-way ANOVA, *N* = 4). (**G** and **H**) Freshly eclosed female (G) and male (H) *ctrl* flies exhibit enhanced survival under starvation conditions, living ~3 days longer than 5-day-old starved *ctrl* flies (age × time interaction: *P* < 0.0001, two-way RM ANOVA, *N* = 3). Data are means ± SEM. *N*, number of independent replicates. For statistical details, see table S1, and for detailed genotype descriptions, see table S2.

To better understand the coordination between excretion and feeding, we examined the chronological relationship between meconium excretion and the initiation of adult feeding in control flies. Because alleles in this study were generated in a *y w* genetic background, flies of a *y w* genetic background were used as “wild-type” and are referred to as control or *ctrl* in the text. We found that meconium deposits, identifiable by their greenish coloration, were excreted in multiple steps over several hours (Fig. 1, A and B). Despite sexually dimorphic traits in feeding (*29*, *30*) and metabolism (*31*–*33*), the dynamics of meconium excretion were comparable between males and females (Fig. 1B).

To examine when adult flies initiate feeding, we kept freshly eclosed flies in a petri dish with a piece of blue-dyed food. Ingested blue-dyed food and its excreta are readily discernible through its accumulation in the abdomen and as blue deposits in the petri dish, respectively. Freshly eclosed flies exposed to blue-dyed food exhibited visible ingestion ~3 hours post-eclosion, with the first blue-dyed excreta appearing after 4 hours (Fig. 1, C and D). Notably, females exhibited an earlier onset of feeding: 100% of females initiated feeding within 24 hours, whereas only ~70% of males had done so (Fig. 1D). Although females have previously been shown to consume more food than males (*29*, *30*), it is nevertheless notable that, despite comparable dynamics of meconium excretion, females initiate feeding earlier. This may reflect previously described sex differences in neuronal and hormonal mechanisms of feeding regulation, such as ecdysone and the response of defined enteric neurons to such hormonal cues (*34*, *35*), and/or a lower threshold for triggering starvation-related responses in females.

To assess how starvation affects meconium excretion, we kept freshly eclosed flies in a petri dish without food, and quantified meconium deposits. In the absence of food, meconium excretion was significantly delayed in both sexes, suggesting that a food-derived cue(s) facilitates the process (Fig. 1, E and F).

Absence of food has other and seemingly more marked consequences for juvenile flies. For instance, oogenesis is suspended at a regulatory checkpoint that prevents the onset of vitellogenesis (*36*, *37*). In addition, the breakdown of energy-storing larval fat cells is delayed by several days (*38*). Without access to food, flies typically survive no longer than 2 to 3 days (*39*). To confirm this, we assessed the survival of freshly eclosed and well-fed 5-day-old control male and female flies. Regardless of sex, 5-day-old flies died within 3 days of starvation (Fig. 1, G and H). Despite never having consumed food, juvenile flies survived up to 3 days longer [Fig. 1, G and H; see also (*38*)].

So far, our observations indicate that in control flies, during the first day of life, the meconium is gradually replaced by ingested food. This suggests a defined temporal sequence between meconium excretion and feeding initiation.

### Meconium retention induces a starvation-like phenotype in *ap* mutant flies

Among thousands of *Drosophila* mutations known so far, *ap* null mutants exhibit a survival pattern that closely resembles starvation-induced lethality in wild-type flies (*18*). The *ap* gene codes for a LIM-homeodomain transcription factor (*40*) and is best known for its role as a dorsal selector gene during wing formation, where it establishes dorsoventral compartmentalization in imaginal wing and haltere discs (*40*–*46*). In addition, Ap plays a role in the development of the embryonic and larval nervous system (*40*, *47*–*49*) as well as in the development of distinct embryonic muscles (*50*). However, very little is known about its function to enable an adult fly to survive longer than 3 to 4 days.

**Fig. 2.**
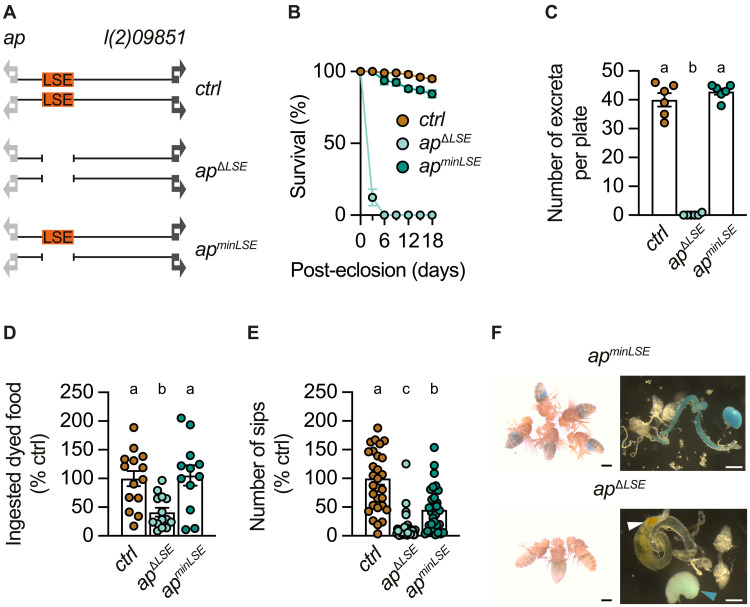
Deletion of the regulatory *apterous* LSE impairs meconium excretion, feeding initiation, and survival of adult flies. (**A**) Schematic representation of the *ap* locus in wild-type (*ctrl*), *ap*^∆*LSE*^, and *ap^minLSE^* flies. *ap*, its flanking gene *l(2)09851*, and the LSE are depicted in light gray, dark gray, and orange, respectively. (**B**) Survival curves of wild-type (*ctrl*), *ap^∆LSE^*, and *ap^minLSE^* mixed-sex flies (time × genotype interaction: *P* < 0.0001, two-way ANOVA, *N* = 4 to 5). (**C**) Total number of excreta of 1-day-old female *ctrl, ap^∆LSE^*, and *ap^minLSE^* flies maintained on blue-dyed food over a period of 2 hours (genotype effect: *P* = 0.0003, Kruskal-Wallis ANOVA, *N* = 6). (**D**) Mean amount of ingested dyed food per fly over 1 hour of feeding in 1-day-old *ctrl*, *ap^∆LSE^*, and *ap^minLSE^* female virgins. Flies were starved overnight, exposed to blue-dyed food the day after and assessed using the squash assay (genotype effect: *P* = 0.0029, Kruskal-Wallis ANOVA, *N* = 12 to 14). (**E**) The relative number of proboscis sips per fly over 1 hour of feeding is reduced in 1-day-old *ap^∆LSE^* female virgins as opposed to *ctrl* and *ap^minLSE^* flies under ad libitum conditions (genotype effect: *P* < 0.0001, Kruskal-Wallis ANOVA, *n* = 30 to 39). (**F**) Images of 1-day-old female *ap^minLSE^* (top) and *ap^∆LSE^* (bottom) flies constantly kept on blue-dyed food and of their guts/crops. In *ap^minLSE^* flies, the blue dye can be detected in crop and midgut. In *ap*^∆*LSE*^ flies, the midgut contains meconium (white arrowhead), and blue dye can only be detected in the crop (blue arrowhead). Data are means ± SEM. *n*, number of flies; *N*, number of technical replicates. Groups that do not differ significantly share the same letter, whereas groups with different letters are statistically significant. For details about statistics and genotypes, see tables S1 and S2.

Through detailed genetic analyses of the *ap* locus, we identified a putative regulatory DNA element referred to as the life-span enhancer (LSE; Fig. 2A and fig. S1A), which is required and sufficient to promote adult survival, but has no function in brain, wing, and haltere development [see (*51*) for a detailed genetic analysis]. We found that over 80% of homozygous *ap* mutant flies, which lack both copies of the LSE but carry intact copies of the *ap* coding region (referred to as *ap*^Δ*LSE*^ flies; Fig. 2A and fig. S1D), die within 3 days post-eclosion as opposed to control flies, which live much longer (Fig. 2, A and B). Reintroducing one copy of the LSE into an LSE-deleted background was sufficient to fully rescue precocious death (referred to as *ap^minLSE^*; Fig. 2, A and B, and fig. S1G). These findings show that the loss of the LSE is responsible for the precocious death phenotype and that *ap*^Δ*LSE*^ mutant flies grown on rich medium exhibited mortality rates similar to those of starved control flies (compare with Fig. 1, G and H).

**Fig. 3.**
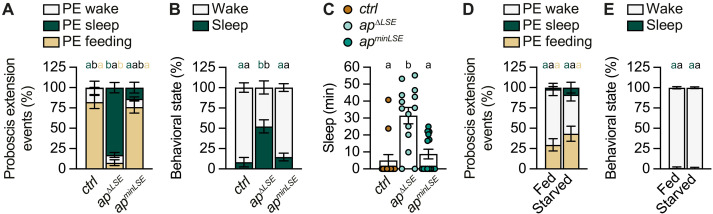
Deletion of the regulatory *apterous* LSE promotes a sleep-like state. (**A**) Annotated PE ratios during wake, sleep, or toward food over a 1-hour recording show that *ctrl* and *ap^minLSE^* flies direct most of their PEs onto food, while *ap*^Δ*LSE*^ flies do so significantly more during sleep (genotype effect: *P* < 0.0001, PERMANOVA, *n* = 13 to 14). (**B**) *ctrl* and *ap^minLSE^* flies spend more time active than sleeping when compared to *ap*^∆*LSE*^ flies over the same 1-hour recording analyzed in (A) (genotype effect: *P* < 0.0001, PERMANOVA, *n* = 13 to 14). (**C**) Total amount of sleep in *ctrl, ap^minLSE^*, *and ap*^Δ*LSE*^ flies over the same 1-hour recording analyzed in (A) and (B) (genotype effect: *P* = 0.0003, Kruskal-Wallis ANOVA, *n* = 13 to 14). (**D**) Annotated PE ratios in fed versus starved flies over a 1-hour recording show that both groups behave similarly (genotype effect: *P* = 0.088, PERMANOVA, *n* = 8 to 12). (**E**) Fed and starved *ctrl* flies do not differ in their percentage of time spent awake or asleep (genotype effect: *P* = 0.0867, PERMANOVA, *n* = 8 to 12). Virgin female flies were used for all experiments. Data are means ± SEM. *n*, number of flies. Groups that do not differ significantly share the same letter, whereas groups with different letters are statistically significant. For statistical details, see table S1, and for detailed genotype descriptions, see table S2.

Further experiments revealed that *ap*^Δ*LSE*^ flies were unable to excrete their meconium and to defecate, in contrast to control and LSE rescue flies (Fig. 2C). Based on our findings regarding the temporal coupling of meconium excretion with feeding initiation (compare Fig. 1), we hypothesized that *ap*^Δ*LSE*^ flies may be unable to properly initiate feeding. One-day-old *ap*^Δ*LSE*^ flies barely ingested any food, with only occasional traces of blue-dyed food observed in the crop but never in the midgut (Fig. 2F and fig. S2, A to E). Moreover, 1-day-old *ap*^Δ*LSE*^ flies also displayed a significant reduction in food interaction events when compared to control and *ap^minLSE^* rescue flies (Fig. 2E), suggesting that their feeding behavior was severely impaired. While excretion and food ingestion of *ap^minLSE^* animals are similar to *ctrl* (Fig. 2, C and D), food interaction is somewhat reduced (Fig. 2E). The reason for this slight haploinsufficiency remains unclear but might be explained by a marginal, although not significant, increase in sleep (Fig. 3, A to C, and fig. S3).

To investigate whether the feeding phenotype of *ap*^Δ*LSE*^ flies might be related to meconium retention, we dissected the midguts of control and mutant flies and assessed their contents. These dissections revealed that *ap*^Δ*LSE*^ flies retained their meconium in their midguts (Fig. 2F) far beyond the expected time of excretion (compare Fig. 1). These results support a link between impaired intestinal clearance and feeding, and indicate that the progression from meconium excretion to feeding initiation is severely disturbed in *ap*^Δ*LSE*^ mutants.

### *ap* mutant flies are engaged in proboscis extension sleep

To gain deeper insights into this temporal window, we conducted high-resolution imaging analyses of freely moving *ctrl* and *ap*^Δ*LSE*^ flies, and quantified their movement as well as different types of proboscis extension (PE) events over a 1-hour experimental period: PE directed toward food, PE during active movement, and PE during sleep. In control and *ap^minLSE^* flies, more than 90% of PE events were associated with food consumption (PE feeding), with only a minor proportion occurring during either locomotion and grooming (PE wake) or sleep (PE sleep). In contrast, *ap*^Δ*LSE*^ mutants predominantly displayed sleep-associated PEs (~80% of total PE events) (Fig. 3A, fig. S3A, and movies S2 to S4). These data indicate that the impaired feeding behavior of *ap*^Δ*LSE*^ flies is not due to proboscis dysfunction. Instead, *ap*^Δ*LSE*^ mutants exhibit a redistribution of PE events across behavioral states.

The periodic occurrence of PE movements during immobility in *ap*^Δ*LSE*^ mutants is highly reminiscent of proboscis extension sleep (PES), a distinct deep sleep state previously described in *Drosophila* (*52*). Unlike stereotypical feeding-associated PEs, PES is characterized by unique dynamics and kinematics (*52*, *53*). Comparative analyses confirmed that the PE events displayed by *ap*^Δ*LSE*^ mutants during immobility closely matched the defining features of PES (fig. S4). In addition, *ap*^Δ*LSE*^ flies exhibited predominantly prolonged immobility bouts of ≥5-min inactivity—an established criterion of sleep in *Drosophila* (*54*)—further supporting the finding that *ap*^Δ*LSE*^ mutants are engaged in PES (Fig. 3, B and C, and figs. S3B and S6). Notably, the sleep phenotypes were fully rescued in *ap^minLSE^* flies (Fig. 3, B and C, and fig. S3B).

To determine whether the PES-like phenotype in *ap*^Δ*LSE*^ flies could be attributed to a starvation-like state, we performed the same assay on 1-day-old control flies subjected to acute starvation immediately post-eclosion. During the 1-hour recording period, during which they had access to agar only, neither fed nor starved control flies showed differences in the distribution of PE events across conditions (Fig. 3D and fig. S5A) and remained highly active throughout the experiment (Fig. 3E and fig. S5B). This suggests that acute starvation alone is not sufficient to induce PES in juvenile control flies. Moreover, in a multiday recording spanning the life span of *ap*^Δ*LSE*^ mutants and conducted in the presence of food, *ap*^Δ*LSE*^ flies showed a marked reduction in overall locomotion activity when compared to control flies (fig. S7). This stands in contrast to the hyperactivity phenotype observed in juvenile starved flies, which has been speculated to facilitate food acquisition and energy intake under scarce food conditions (fig. S7) (*55*). To determine whether *ap*^Δ*LSE*^ mutants exhibit a metabolic response to starvation, we measured the accumulation of insulin-like peptide 2 (Ilp2) in insulin-producing cells (IPCs), a well-established readout of nutrient availability (*56*). Ilp2 accumulated in the IPCs of *ap*^Δ*LSE*^ mutants, as in starved wild-type flies, at significantly higher levels than in fed controls (fig. S8, A and B). Together, these results indicate that *ap*^Δ*LSE*^ mutants are in a metabolically starved state but fail to respond to it by, e.g., seeking food intake, and instead predominantly enter a PES state.

### The LSE mediates *ap* expression in the posterior hindgut

**Fig. 4.**
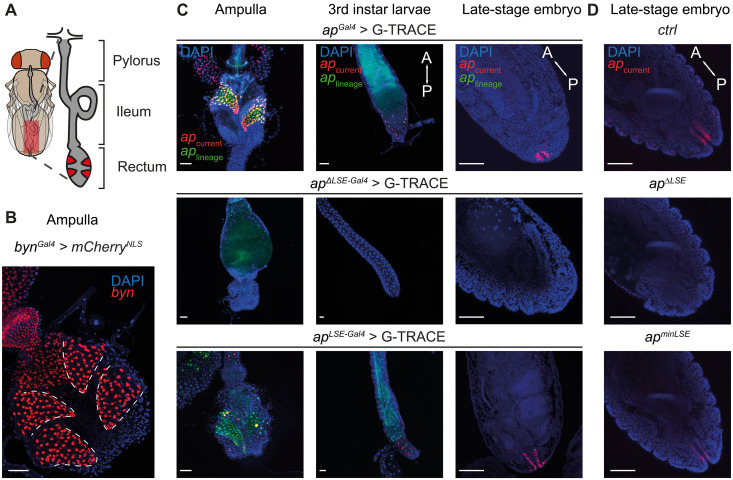
The *apterous* LSE is expressed in the posterior hindgut during all developmental stages. (**A**) Illustration of the posterior hindgut of adult *Drosophila*. Rectal papillae (red), responsible for reabsorption processes from primary urine, are located in the rectum. (**B**) A *Gal4* reporter for the gene *brachyenteron* (*byn*) serves as a robust marker for hindgut cells including rectal papillae (all four papillae are visible). (**C**) G-TRACE fluorescence analysis of *apterous Gal4* driver lines focusing on the posterior hindgut across developmental stages. Fluorescence images reveal *ap* expression in the rectal papillae and hindgut cells during adult, larval, and embryonic stages in *ap^Gal4^* and *ap^LSE-Gal4^* but not *ap*^∆*LSE-Gal4*^ flies. This shows that the LSE is required and sufficient for hindgut-specific *ap* expression throughout development. Blue, DAPI; red and green, current and cell lineage expression of *ap Gal4* driver lines, respectively. (**D**) Tissue specificity of *ap*^*Gal4*^ driver lines shown by Ap antibody stainings of posterior hindguts in late-stage embryos. Mix-sexed flies were used for all experiments except in (B), in which a virgin female fly was used. Blue, DAPI; red, Ap detected using anti-Ap antibody. A, anterior, P, posterior. Scale bars, 50 μm. For detailed genotype descriptions, see table S2.

In light of our observation that *ap*^Δ*LSE*^ mutants fail to excrete their meconium, these findings raise the possibility that impaired intestinal clearance, rather than starvation per se, may promote the prolonged sleep-like state and the broader physiological and behavioral alterations observed in these mutants. To understand how the LSE deletion triggered the feeding, excretion, and sleep phenotypes, we next investigated how and where this deletion affected *ap* expression. Ap is dynamically expressed throughout development in various tissues, for which several tissue-specific enhancers have been identified (*40*, *44*, *45*, *48*, *50*, *57*–*60*). We hypothesized that our genetically characterized LSE (*51*) corresponds to a tissue-specific regulatory element that activates the gene in a subset of Ap-expressing cells. To determine the anatomical site of action of the LSE, we engineered enhancer-trap–like *Gal4* driver lines in the *ap* locus with (*ap^Gal4^* and *ap^LSE-Gal4^*; fig. S1, H and J) or without (*ap*^Δ*LSE-Gal4*^ and *ap*^Δ*LSE-Gal4_2*^; fig. S1, I and K) regulation by LSE, and traced their cell lineage using the G-TRACE system. G-TRACE tracks all cells that currently express Gal4 (*ap*_current_) or have done so in the past (*ap*_cell lineage_) when activated by a *Gal4* driver (*61*). Notably, the analysis of the Ap expression patterns revealed that the LSE was active in the posterior hindgut (Fig. 4). This finding is of broad interest given the observed feeding and excretion phenotypes of *ap* mutants.

The hindgut consists of three subdivisions: pylorus, ileum, and rectum [Fig. 4A; reviewed in (*62*)]. A key structure within the adult rectum is the ampulla, which contains four cone-shaped rectal papillae that can be visualized using the *brachyenteron* [*byn*; (*63*)] *Gal4* driver [*byn^Gal4^*; (*64*)] (Fig. 4B). Together with the malpighian tubules, rectal papillae function analogously to mammalian kidneys [reviewed in (*62*)]. We found that LSE-driven *ap* expression was restricted to two of the four rectal papillae and that this signal was completely absent in the hindguts of LSE-deleted (*ap*^Δ*LSE-Gal4*^) flies (Fig. 4C). Notably, our developmental G-TRACE analysis further revealed that *ap* was already expressed in the posterior hindgut of the embryo, where it labels two stripes that are absent in *ap*^Δ*LSE-Gal4*^ flies (Fig. 3C and movie S1). Immunostaining of Ap in *ctrl*, *ap*^Δ*LSE*^, and *ap^minLSE^* embryos confirmed the G-TRACE expression patterns (Fig. 4D), validating the newly established *Gal4* drivers as reliable reporters of Ap expression (see also figs. S12 and S13). These findings show that LSE activates *ap* expression in the posterior hindgut as early as embryonic stages and that *ap* expression persists in two of the four rectal papillae in adult flies.

### An ileus-like physical obstruction is present in the hindgut of *ap*^∆*LSE*^ flies

**Fig. 5.**
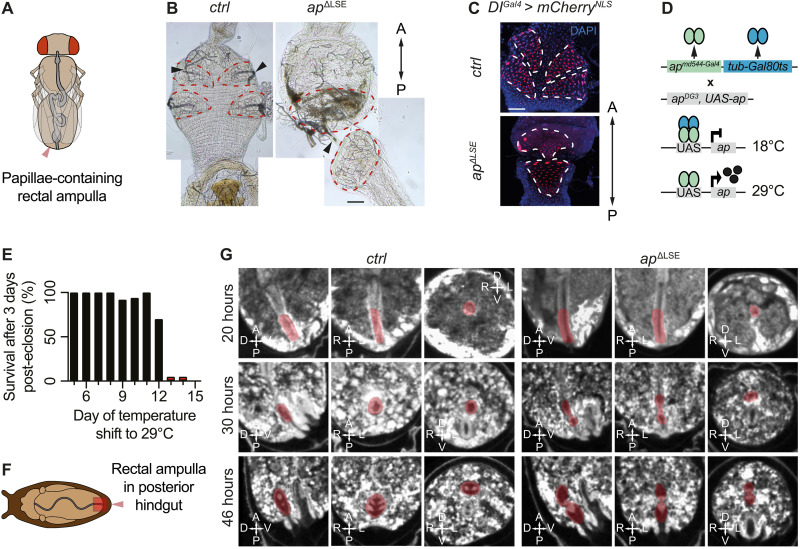
The hindgut-obstructing Reinger’s knot is formed in the absence of the *apterous* LSE. (**A**) Schematic overview of an adult fly with ampulla containing four rectal papillae (red arrow). (**B**) The rectal papillae (red outlines) of *ctrl* flies contain an extensive tracheal network (black arrowheads). In *ap*^*∆LSE*^ mutants, the arrowhead points to a narrow duct connecting the anterior and posterior parts of the Reinger's knot (red outline). (**C**) Top: *Dl*^Gal4^** expression in rectal papilla cells; *mCherry^NLS^* (red) marks all four papillae (white outlines). Bottom: Reinger’s (white outline) knot in *ap*^∆*LSE*^ mutants also expresses *Dl^*Gal4*^* (red). (**D**) *tubGal80^ts^* temperature shift experiments were performed to determine the *ap*-dependent window for papillae formation. *ap^md544^* (*Gal4* driver and strong *ap* allele) (green) recombined with *tubGal80^ts^* (blue) was crossed with *ap^DG3^ UAS-ap*, which lacks the LSE (gray). At 18°C, Ap protein is not expressed; at 29°C, Gal80^ts^ is inactive and Ap is expressed. (**E**) Only flies shifted to 29°C before day 13 survived normally (black bars) (*n* = 10 to 20; see table S3). (**F**) Schematic of a pupa (light brown) in its case (dark brown), showing the intestine (gray) and the posterior hindgut with the rectal ampulla highlighted in red. (**G**) μ-CT scans of papillae formation in *ctrl* and *ap*^∆*LSE*^ pupae at 20, 30, and 46 hours apf. At 20 hours, the ampulla (highlighted in red) appears similar. By 30 hours, *ctrl* flies show dorsal and ventral papilla regions, while *ap*^∆*LSE*^ flies show a bulbous tissue structure within the ampulla. By 46 hours, *ctrl* flies form four papillae, and *ap*^∆*LSE*^ flies form the Reinger’s knot. Virgin female flies were used for all experiments. D, dorsal; V, ventral; L, left; R, right. Scale bars, 50 μm. For genotypes, see table S2.

To determine whether the absence of *ap* expression in the hindgut of *ap*^Δ*LSE*^ mutants might affect rectal papillae formation, we examined dissected ampullae from 1-day-old adult *ap*^Δ*LSE*^ flies using wide-field and confocal imaging. In control flies, four papillae per ampulla are formed, each targeted by an intricate tracheal network (Fig. 5, A and B) (*65*). In *ap*^Δ*LSE*^ flies, papillae were absent, and, instead, a malformed structure, which we term the Reinger’s knot, formed at the posterior end of the ampulla (Fig. 5B). This structure appeared to consist of mispositioned papilla cells that failed to separate and properly integrate in the ampulla, as indicated by persistent *Delta*^*Gal4*^ (*Dl*^*Gal4*^) expression, a marker for rectal papilla cells (Fig. 5C) (*66*). Note that the Reinger’s knot is still targeted by a tracheal jumble. These imaging experiments show that the rectal papillae of adult *ap*^Δ*LSE*^ flies fail to form properly and also suggest that the Reinger’s knot fully obstructs the hindgut, potentially explaining why *ap*^Δ*LSE*^ flies are unable to excrete their meconium. This occlusion functionally resembles an ileus in humans, a condition that, if untreated, is lethal (*67*–*69*). Note that the hindgut posterior to the Reinger’s knot is not obstructed in *ap*^Δ*LSE*^ flies (fig. S9).

To identify the developmental stage at which *ap* is required for rectal papillae formation, we performed temperature-sensitive rescue experiments using the Gal4/Gal80^ts^/upstream activating sequence (UAS) system to restore *ap* expression in an otherwise mutant background (Fig. 4D). We found that Ap protein expression must be initiated before day 13 after egg laying (AEL) at 18°C (which corresponds to early metamorphosis) to rescue precocious lethality (Fig. 5E and table S3) (*70*). This suggests that *ap* may play a key role in rectal papillae formation during early metamorphosis [see also (*71*)].

To test this, we performed micro–computed tomography (μ-CT) imaging (*72*, *73*) on 20-, 30-, and 46-hour-old control and *ap*^Δ*LSE*^ pupae to visualize internal structures throughout development (Fig. 5, F and G). Differences in hindgut morphology first appeared at 30 hours after pupa formation (apf) with rectal papillae forming at the ampulla’s edges of control flies, while remaining centrally positioned in *ap*^Δ*LSE*^ mutants. By 46 hours apf, control pupae had four distinct papillae, whereas *ap*^Δ*LSE*^ mutants retained a central mass obstructing the hindgut (Fig. 5G). This suggests that papillae formation is arrested at the stage when the rectal tube normally splits into four cone-shaped luminal structures (*74*).

The three-dimensional (3D) visualization of the entire gut of 1-day-old control, *ap*^Δ*LSE*^, and *ap^minLSE^* flies revealed an additional phenotype in the midgut. While control and *ap^minLSE^* midguts appeared normal, the midgut was bloated in *ap*^Δ*LSE*^ flies (fig. S10A). Actin immunostaining showed no structural abnormalities in the visceral mesoderm of *ap*^Δ*LSE*^ flies (fig. S10C), but by day 2, *ap*^Δ*LSE*^ midguts frequently showed signs of decay (fig. S10D), suggesting rupture and leakage into the abdominal cavity. In summary, our analysis of adult gut morphology in control and *ap*^Δ*LSE*^ flies reveals the formation of the Reinger’s knot during early metamorphosis and subsequent midgut deterioration in 2-day-old *ap*^Δ*LSE*^ adults.

### Ap is only required during early metamorphosis for normal papillae formation

**Fig. 6.**
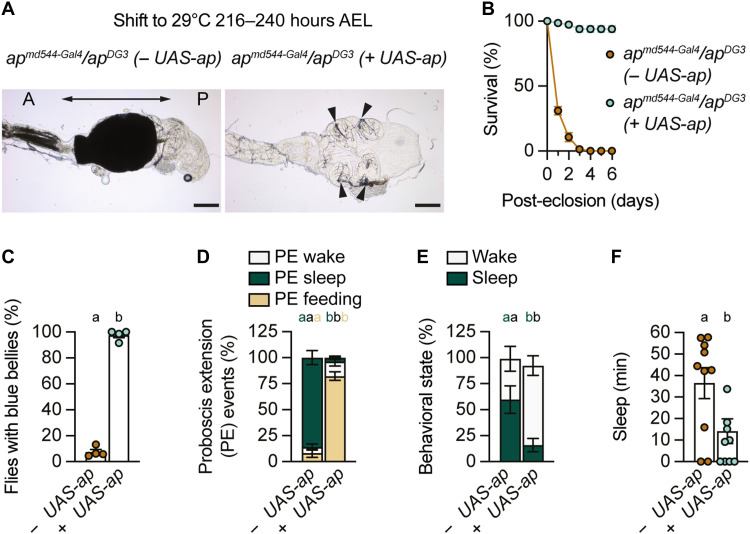
Papilla formation depends on *ap* early during metamorphosis. (**A**) A *tubGal80^ts^*-based 48-hour pulse of Ap expression applied 216 to 240 hours AEL is sufficient to rescue papillae development in the presence of *UAS-ap* in an *ap^md544^/ap^DG3^* mutant background (see also Fig. 5D). In the absence of *UAS-ap*, the flies display the Reinger’s knot. (**B** to **F**) Pulsed expression of Ap as detailed in (A) is sufficient to rescue survival (B), feeding initiation (C), PE ratios (D), percent time spent awake or asleep (E), and total sleep duration (F). (B) Pulse effect: *P* < 0.0001; time × pulse interaction: *P* < 0.0001, two-way ANOVA, *N* = 4. (C) Genotype effect: *P* = 0.0286, Mann-Whitney test, *N* = 4. (D) Genotype effect: *P* < 0.0001, PERMANOVA, *n* = 9 to 10. (E) Genotype effect: *P* = 0.006, PERMANOVA, *n* = 9 to 10. (F) Genotype effect: *P* = 0.0144, Mann-Whitney test, *n* = 9 to 10. Virgin female flies were used for all experiments. Data are means ± SEM. *n*, number of flies; *N*, number of technical replicates. Groups that do not differ significantly share the same letter, whereas groups with different letters are statistically significant. For statistical details, see table S1, and for detailed genotype descriptions see table S2. Scale bars, 50 μm.

To determine whether transient Ap expression in the hindgut is sufficient to rescue the morphological and behavioral phenotypes of *ap*^Δ*LSE*^ mutants, we performed a temperature shift pulse experiment using the Gal4/Gal80^ts^/UAS system. A 48-hour exposure of late third instar larvae to 29°C was sufficient to rescue papillae formation in the presence but not in the absence of the *UAS-ap* transgene (Fig. 6A). Notably, the transient hindgut-specific expression of Ap also rescued precocious adult lethality (Fig. 6B), restored feeding initiation (Fig. 6C), and markedly reduced PES (Fig. 6, D and E, and fig. S11A) and overall sleep amount (Fig. 6F and fig. S11B).

In addition to its expression in the hindgut, *ap* is also expressed in the adult brain, raising the possibility that *ap* might influence feeding and sleep through Ap-expressing cells in the brain. To examine this possibility, we used an additional ap^Δ*LSE-Gal4*^ enhancer-trap driver line (referred to as *ap*^Δ*LSE-Gal4_2*^; see fig. S1K), which lacks the LSE, and therefore Ap hindgut expression, but contains an intact central nervous system enhancer (*apS* in fig. S1A) (*49*, *60*). The *ap*^Δ*LSE-Gal4_2*^-driven CD8::GFP (green fluorescent protein) expression patterns in embryonic and adult brains were very similar to those observed for *ap^Gal4^* (fig. S12, A and B). Notably, Ap expression in *ap*^Δ*LSE-Gal4_2*^ flies was not sufficient to rescue the Reinger’s knot (fig. S13), survival (figs. S12C and S13A), feeding initiation (fig. S12D), excretion (fig. S12E), PES (figs. S12F and S14, A and B), as well as total sleep amount (figs. S12, G and H, and S14, A and B). This was in contrast to the results obtained with *ap^Gal4^* driver lines, which contain the LSE and therefore promote Ap hindgut expression (figs. S12 and S13).

Together, these findings show that loss of Ap expression in the hindgut disrupts rectal papillae formation during metamorphosis, resulting in the formation of the Reinger’s knot and impaired adult physiological functions. In contrast, limiting Ap expression mostly to the hindgut is sufficient to rescue gut morphology, survival, feeding, and sleep phenotypes in *ap^LSE-Gal4^* flies.

### Removal of the Reinger’s knot restores survival in *ap*^𝚫*LSE*^ flies

**Fig. 7.**
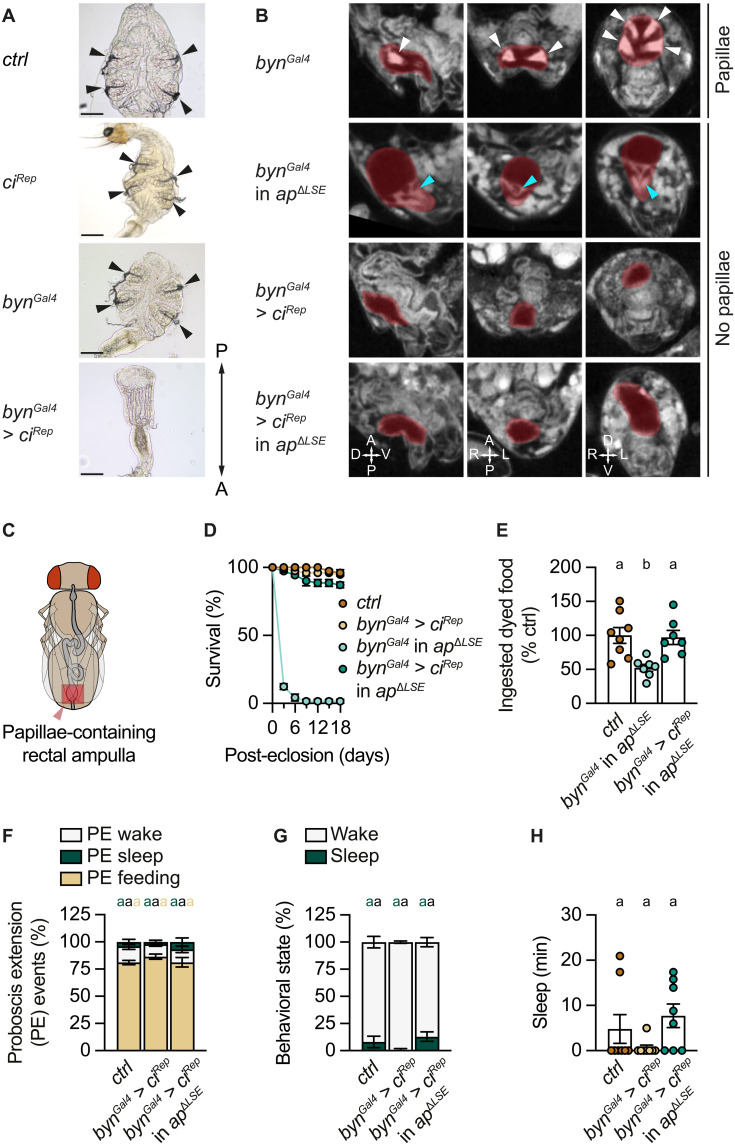
The removal of the Reinger’s knot rescues the excretion, feeding, sleep, and survival phenotypes of *ap*^𝚫*LSE*^ flies. (**A**) Four papillae are found in the rectal ampulla of *ctrl*, *ci^Rep^*, and *byn^Gal4^* flies (black arrowheads) but are absent in *byn^Gal4^* > *ci^Rep^* flies. (**B**) μ-CT scans show that papillae (highlighted in red, white arrowheads) are found in *byn^Gal4^* flies in a wild-type but not *ap^∆LSE^* mutant background. *byn^Gal4^* > *ci^Rep^* eliminates the formation of normal papillae in a wild-type background, and of the Reinger’s knot in an *ap^∆LSE^* mutant background (highlighted in red, blue arrowheads). (**C**) Schematic overview of an adult fly indicating the position of the ampulla (highlighted in red) within the intestine (gray). (**D** to **H**) Removal of the Reinger’s knot using *byn^Gal4^* > *ci^Rep^* is sufficient to rescue survival (D), feeding initiation (E), PE ratios (F), percent time spent awake or asleep (G), and total sleep duration (H). (D) Genotype effect: *P* < 0.0001; time × genotype interaction: *P* < 0.0001, two-way ANOVA, *N* = 3. (E) Genotype effect: *P* = 0.0012, Kruskal-Wallis ANOVA, *N* = 7 to 8. (F) Genotype effect: *P* = 0.543, PERMANOVA, *n* = 8. (G) Genotype effect: *P* = 0.132, PERMANOVA, *n* = 8. (H) Genotype effect: *P* = 0.1175, Kruskal-Wallis ANOVA, *n* = 8. Virgin female flies were used for all experiments except in (D), in which mixed-sex flies were used. Data are means ± SEM. *n*, number of flies; *N*, number of technical replicates. Groups that do not differ significantly share the same letter, whereas groups with different letters are statistically significant. For statistical details, see table S1, and for detailed genotype descriptions, see table S2. Scale bars, 50 μm.

To assess whether the Reinger’s knot and/or midgut deterioration is responsible for the precocious adult death phenotype, we sought to prevent hindgut obstruction in *ap*^Δ*LSE*^ flies by eliminating the Reinger’s knot. Since we found that the Reinger’s knot consists of mislocalized Dl-expressing papilla cells, we hypothesized that the knot could eventually be removed by eliminating these cells. Flies lacking rectal papillae have been previously obtained by manipulating the cohesin complex required for mitotic fidelity of polyploid papillar cells (*75*). Another study on hindgut proliferation suggested that expressing the repressor isoform of *cubitus interruptus* [*ci^Rep^*; (*76*, *77*)] in the posterior hindgut using *byn^Gal4^* might prevent rectal papillae formation. However, how *byn^Gal4^* > *ci^Rep^* interferes with papillar development is not known [see figure 4C in (*78*)]. *byn^Gal4^* is exclusively active in the hindgut (Fig. 4B and fig. S12B) but not in the brain (fig. S12, A and B). We replicated the experiment of Takashima *et al.* (*78*) and confirmed that *byn^Gal4^* > *ci^Rep^* flies completely lacked rectal papillae (Fig. 7A), providing a means to ablate these cells. Notably, these flies remained viable under standard laboratory conditions but perished within 9 days when raised on high-salt medium (250 mM; fig. S15A).

To determine whether *ci^Rep^* expression in the hindgut disrupts rectal papillae formation during a developmental time window similar to that of *ap*, we performed temperature-shift experiments using the tub-Gal80^ts^ system (*70*). Our findings indicate that *ci^Rep^* acts at the onset of metamorphosis, ~1 day before *ap* is required (fig. S15, B to D, compare to Fig. 5E; table S4; see legends for details).

To evaluate the effect of papilla removal on Reinger’s knot formation, we expressed *ci^Rep^* under *byn^Gal4^* control in *ap*^Δ*LSE*^ mutants. μ-CT imaging confirmed the absence of rectal papillae and the elimination of the Reinger’s knot in adult flies (Fig. 7B), demonstrating that *byn^Gal4^* > *ci^Rep^* disrupts papillae formation upstream of *ap*. μ-CT scans also showed that the midguts of the knot-ablated *ap*^Δ*LSE*^ mutants were normal (fig. S10B), strongly indicating that the Reinger’s knot is the primary cause of the bloated appearance and subsequent tissue decay observed in the midguts of *ap*^Δ*LSE*^ flies (fig. S10D). Notably, knot-ablated *ap*^Δ*LSE*^ mutants were viable (Fig. 7D) and fertile. They also exhibited restored feeding behavior (Fig. 7E), activity, and sleep levels (Fig. 7, F to H, and fig. S16B) and showed no signs of PES (Fig. 7F and fig. S16A) when compared to control flies as well as parental controls (figs. S17 and S18). Together, these findings indicate that hindgut blockade (via the Reinger’s knot) is a major contributor to the survival, feeding, and activity/sleep phenotypes observed in *ap*^Δ*LSE*^ mutants, and underscores the critical role of *ap* in maintaining normal behavior in adult flies.

### Meconium retention by external obstruction induces precocious adult death

**Fig. 8.**
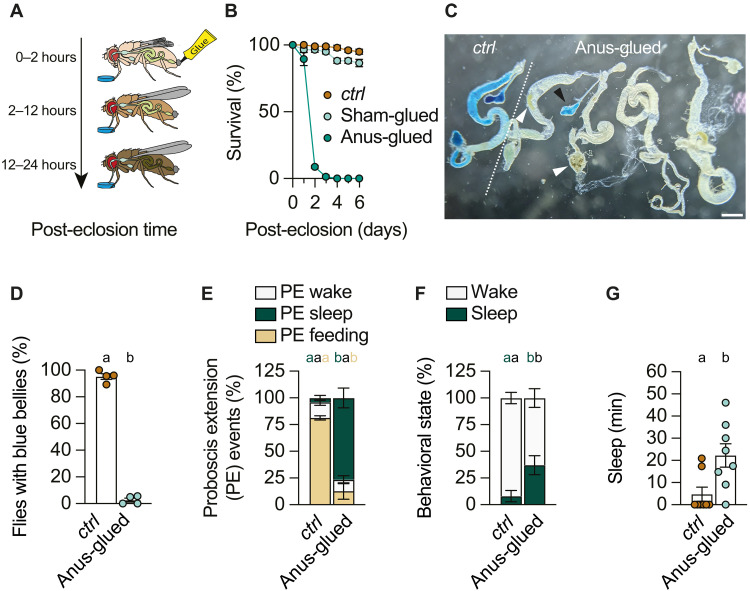
Sealing the anus of wild-type flies phenocopies the behavior of *ap*^𝚫*LSE*^ mutants. (**A**) Schematic overview of the sealed-anus experiment. Freshly eclosed flies were briefly anesthetized to apply a drop of glue to their anus to prevent meconium excretion. As a result, flies with a sealed anus did not initiate feeding and, as observed for *ap*^∆*LSE*^ flies, developed a darker appearance. (**B**) Sealing the anus decreases survival when compared to both intact and sham-glued control groups (phenotype effect: *P* < 0.0001; time × phenotype interaction: *P* < 0.0001, two-way ANOVA, *N* = 3 to 4). (**C**) Like in *ap*^∆*LSE*^ flies, *ctrl* flies with a sealed anus fail to initiate adult feeding as indicated by the absence of ingested blue-dyed food in dissected 1-day-old intestines. Blue food can enter the crop (black arrowhead) but never the midgut (see also fig. S2). Meconium leftovers (white arrowheads) can be detected within the intestine. (**D**) Sealing the anus prevents feeding initiation when compared to *ctrl* flies (genotype effect: *P* = 0.0286, Mann-Whitney test, *N* = 4). (**E**) Sealing the anus induces a shift in PE ratios, away from food-direct toward sleep-associated (genotype effect: *P* = 0.0002, PERMANOVA, *n* = 8). (**F** and **G**) Anus-glued flies spend significantly more time asleep. (F) Genotype effect: *P* = 0.0104, PERMANOVA, *n* = 8. (G) Genotype effect: *P* = 0.0145, Mann-Whitney test, *n* = 8. Virgin female flies were used for all experiments except in (B), in which mixed-sex flies were used. Data are means ± SEM. *n*, number of flies; *N*, number of technical replicates. Groups that do not differ significantly share the same letter, whereas groups with different letters are statistically significant. For statistical details, see table S1, and for detailed genotype descriptions, see table S2. Scale bar, 200 μm.

If intestinal clearance, including meconium excretion, contributes to feeding initiation, then artificially blocking the anus of newly eclosed wild-type flies should produce opposing effects and mimic the *ap*^Δ*LSE*^ phenotype. To test this, we applied superglue to the anus (anus-glued) or to the anterior abdomen (sham-glued) as a control (Fig. 8A). While application of superglue to the anterior abdomen had no adverse effects (Fig. 8B), obstructing the anus induced symptoms closely resembling those of *ap*^Δ*LSE*^ mutants. Anus-glued flies exhibited a sharp decline in survival, with ~80% perishing within 3 days (Fig. 8B). Feeding was also impaired, and blue-dyed food was never detected in the midgut but occasionally in the crop (Fig. 8, C and D, and fig. S2). Anus-sealed flies also showed a significant reduction in activity and spent most of their time in PES (Fig. 8, E to G, and fig. S19, A and B).

Together, these results show that external intestinal obstruction, which prevents meconium excretion and retains intestinal contents, is sufficient to impair feeding and to induce a PES state as well as precocious adult death. These findings highlight a previously unrecognized link between intestinal blockade, meconium excretion, feeding initiation, sleep, and survival. However, because anal sealing induces a complete intestinal blockage, these phenotypic effects may reflect a broader physiological dysfunction caused by obstruction rather than a specific consequence of impaired meconium excretion.

## DISCUSSION

**Fig. 9.**
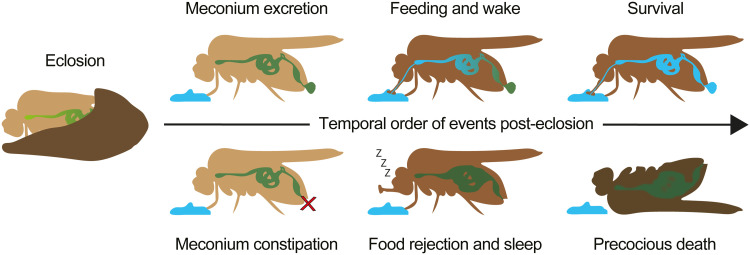
Sequence of events during the short adult life of *ap*^𝚫*LSE*^ mutants. The first 2 days of a *ctrl* (top) or *ap*^∆*LSE*^ fly (bottom) are shown. After eclosion (left), the apparent behavior of *ctrl* and *ap*^∆*LSE*^ flies is indistinguishable during the first few hours of adult life, and both genotypes are equally agile and actively explore their environment. The first indication of their different physiological constitution is the complete lack of excretion in *ap*^∆*LSE*^ flies. A few hours later, blue food is detectable in both the crop and midgut of control flies, whereas in *ap*^∆*LSE*^ flies, it is observed in the crop but never in the midgut. This indicates that feeding per se is not defective, but that the inability of meconium excretion prevents further food ingestion. However, it is unclear whether passage of food to the midgut is not possible simply due to the lack of space or whether obstruction of meconium excretion is instructive for feeding (e.g., by activating certain neuronal circuits). After 24 hours, behavioral differences between *ctrl* and *ap*^∆*LSE*^ flies are blatant. Complete constipation forces *ap*^∆*LSE*^ flies into a survival mode. As previously reported for *ap^null^* flies, the most obvious repercussions of this systemic illness are failure to induce vitellogenesis and delayed larval fat cell histolysis, but the most obvious anomaly is their lethargy. They barely move and frequently remain in PES. Precocious adult death of *ap*^∆*LSE*^ flies most likely has at least two reasons: lack of metabolic input (their death rate is very similar to that of starved *ctrl* flies) and decay of bloated midgut. It also points to a notable analogy in the sequence of events between the systemic illness of *ap*^∆*LSE*^ flies and ileus disease in humans. Both are initiated by an intestinal obstruction, which prevents feces excretion.

Our study identifies a previously uncharacterized developmental and physiological process in *D. melanogaster*: a temporal coupling between meconium excretion and the initiation of adult feeding. We show that the successful transition to post-eclosion feeding is temporally linked to the partial expulsion of the meconium. In addition, we provide a genetic framework underlying the meconium-induced constipation of *ap*^∆*LSE*^ flies, which results in a starvation-like, lethargic phenotype coupled with bouts of PES (Fig. 9). Notably, experimental sealing of the anus in *ctrl* flies phenocopied the behavioral and physiological impairments observed in *ap^∆LSE^* mutants, reinforcing the importance of intestinal clearance for normal feeding. However, our data do not allow us to distinguish whether the observed effects on feeding and sleep arise directly from meconium excretion or indirectly from the consequences of intestinal blockage. Accordingly, it remains unclear whether these processes are functionally coupled through a specific mechanism or whether they reflect broader effects of impaired intestinal clearance.

### Resolving a 110-year-old enigma: *ap* and the precocious adult death phenotype

In 1914, Metz reported on the first *ap* allele (*ap^1^*). It was characterized by pleiotropic phenotypes such as the absence of wings and halteres, premature adult death, and female sterility (*18*). While the role of *ap* in wing development has been extensively studied [reviewed in (*79*)], the mechanisms underlying the precocious adult death phenotype remained unclear. In this study, we describe the role of a cis-regulatory enhancer, the LSE. It is critical for adult survival by ensuring proper rectal papillae formation [see also (*51*)]. In its absence, papilla precursors fail to undergo proper development, leading to the formation of the Reinger’s knot, a hindgut obstruction that ultimately prevents meconium excretion, disrupts adult feeding, and promotes a PES state.

Our findings indicate that *ap* plays a critical role in the morphogenesis of rectal papillae during early pupal development. Our observations are in agreement with earlier studies highlighting its role in hindgut development (*71*, *80*). Notably, the timing of papillae formation coincides with two rounds of mitotic division in the polyploid papilla precursor cells (Fig. 4) (*66*, *74*). In *ap*^Δ*LSE*^ flies, these precursor cells remain clustered together within the Reinger’s knot. Given that the LSE drives *ap* expression in only two of the four rectal papillae, we hypothesize that *ap* regulates the expression of cell surface proteins required for the separation of the papilla precursors into four papillae. This might be similar to the role of *ap* in wing compartmentalization, where it contributes to the separation of dorsal and ventral wing cells (*81*). While Notch signaling is also known to be essential for papillae formation (*66*, *74*), the distinct phenotypes observed in Notch–RNA interference and *ap*^Δ*LSE*^ flies suggest that they play distinct roles. Future studies shall investigate the specific molecular interactions between *ap* and *Notch* signaling in the context of rectal papillae development.

We also found that female sterility, which is observed in all strong *ap* alleles due to a block of vitellogenesis (*18*, *41*, *82*–*84*), is a secondary effect of the conditions induced by the Reinger’s knot in *ap*^∆*LSE*^ flies (*51*). In addition, previous reports have shown that larval fat cell histolysis during early adulthood is delayed in strong *ap* alleles displaying the precocious adult death phenotype (*19*, *20*). Lack of vitellogenesis as well as delayed larval fat cell histolysis can be ameliorated by juvenile hormone treatment (*84*, *85*). Thus, it seems likely that *ap*^Δ*LSE*^ flies respond to their condition by preventing energy-consuming developmental processes. This highlights the fact that under stressful metabolic conditions, flies make critical metabolic decisions on whether to bear the cost of reproduction at the possible expense of survival (*26*, *37*).

The wide range of phenotypes observed in *ap*^Δ*LSE*^ flies indicates that the physiology of multiple organs is affected. Although the complete intestinal blockage caused by the Reinger’s knot is likely at the root of all dysfunctions [also referred to as the “apterous syndrome” in (*51*)], it remains unclear to what extent individual factors contribute to impaired feeding and what molecular mechanisms underlie these effects. In particular, intestinal obstruction is expected to cause accumulation of waste products, tissue stress, and systemic physiological dysfunction, all of which could independently impair feeding behavior. Therefore, while our data are consistent with a role for meconium retention in this process, we cannot exclude the possibility that the observed feeding defects arise from more general consequences of intestinal blockage rather than from a specific requirement for meconium excretion per se.

### A *Drosophila* model for ileus disease: Parallels with human gastrointestinal dysfunction

In human medicine, ileus is defined as a temporary cessation of intestinal peristalsis (*67*). In practice, a distinction is made between mechanical and paralytic ileus. A paralytic ileus can have various pathological causes that lead to an impairment of intestinal peristalsis. In contrast, intestinal peristalsis is not impaired in mechanical ileus, at least initially. Rather, it is the result of a mechanical obstacle to passage. Regardless of its etiology, a first pathophysiological indication of a mechanical ileus is a palpable dilatation of the intestinal wall due to a several fold increase in intraluminal pressure. A second important diagnostic feature of mechanical ileus is hyperperistalsis, by which the intestine attempts to push its contents past the intestinal obstruction. Within the first 24 hours, the mechanical stress leads to a considerable reduction in water and salt ion absorption from the intestinal lumen. In addition, the reduced blood circulation leads to hypoxia and paralysis of the energy-consuming molecular transport systems. A vicious circle is set in motion that further increases the intraluminal pressure. The intestinal epithelium becomes permeable, leading to bleeding, peritonitis, and translocation of intestinal bacteria into the body cavities. An untreated ileus inevitably leads to perforation of the intestinal wall with fatal and life-threatening consequences for the patient (*68*, *69*).

Our detailed analyses of the phenotypes detected in *ap*^Δ*LSE*^ flies suggest that there are some notable parallels to humans suffering from a mechanical ileus. In both flies and humans, reduced appetite, absolute constipation, and intestinal bloating are the first indicators for the pathophysiological condition. Another important diagnostic feature for a mechanical ileus is hyperperistalsis, a compensatory mechanism that attempts to force intestinal contents past the obstruction, eventually leading to increased intraluminal pressure and, if unresolved, tissue rupture with fatal consequences for the patient (*68*, *69*). It will be interesting to investigate possible links between peristalsis and PES in *Drosophila*; it is conceivable that, as well as minimizing energy consumption and increasing hemolymph circulation, it might stimulate gut peristalsis to expel the obstructing meconium. This may explain why starvation in flies that were previously fed does not trigger PES. These findings establish *Drosophila* as a genetically tractable model for investigating ileus-like conditions and physiological aspects of gastrointestinal dysfunction.

Our study not only resolves a century-old mystery surrounding the adult precocious death phenotype in *ap* mutants, but also provides fundamental insights between the coupling of intestinal clearance, feeding initiation, and PES in *D. melanogaster*. Moreover, the identification of a genetic mechanism underlying hindgut obstruction (and, as a consequence, meconium retention) in *ap*^Δ*LSE*^ mutants establishes a tractable model for studying the physiological consequences of intestinal blockage in *Drosophila*. However, our findings do not allow us to disentangle the specific contribution of meconium retention from the broader effects of intestinal obstruction. The accumulation of intestinal contents is likely to induce multiple forms of physiological stresses, including mechanical tension, metabolic imbalance, and systemic dysfunction, any of which could contribute to impaired feeding and/or sleep behavior. Thus, while our data are consistent with a link between the failure to clear developmental waste and impaired feeding initiation, they do not exclude the possibility that these phenotypes arise from more general consequences of gut obstruction. One possibility is that retained intestinal contents, including meconium, generate physiological or mechanical signals that modulate feeding behavior, for example, by mimicking satiety or signaling internal distress. Elucidating how the intestinal state is sensed and communicated to feeding circuits will be an important goal for future studies.

## MATERIALS AND METHODS

### *Drosophila* strains and culture

Flies were housed in a 25°C incubator with 60% humidity under a 12-hour light:12-hour dark cycle and reared on a standard cornmeal agar diet composed of 0.07% (w/v) granulated agar-agar, 0.1% (w/v) cornmeal, 0.06% (w/v) granulated sugar, 0.015% (w/v) granulated dry yeast, and 0.003% (w/v) granulated Nipagin. The solidified medium was supplemented with a drop of fresh baker’s yeast unless otherwise stated. *ap^DG1^*, also referred to as *ap^DG^*, was described in (*58*). *ap^c1.4b^*, *ap^R1^* (also called *ap*^*attP*∆*Enh*^), and *ap^DG3^* are described in (*45*). *ap^R2^* is described in (*46*). *P{UAS-mCherry^NLS^}* is described in (*86*). *UAS-ci^Rep^* (*P{UAS-ci^76^}*) was obtained from P. Thérond [Institut de Biologie Valrose; (*76*)]; *byn^Gal4^* was shared by D. T Fox [Duke University; (*64*)], *y M{vas-int.Dm}zh-2A w* by J. Bischof [University of Zürich; (*87*)], and *P{UAS-ap}* by M. Milán [IRB Barcelona; (*88*)]. The following stocks were obtained from the Bloomington *Drosophila* Stock Center: *G-TRACE* (no. 28281), *ap^md544^* (no. 3041), *P{tub-Gal80^ts^}* (no. 7019), *P{UAS-mCD8::GFP}* (no. 32186), *Dl^Gal^* (no. 67047), and *M{vas-Cas9}ZH-2A* (no. 51323). *PBac{RB}e01573* was purchased from Exelixis (Harvard; no longer available). All apterous alleles generated and used in this study are in a *y w* background. Therefore, *y w* flies were used as wild-type control flies for all experiments. For embryonic immunostaining experiments and survival assays, both female and male flies were used and pooled in the figures unless otherwise specified. For all other experiments, virgin female flies were used. For the construction of *byn^Gal4^ UAS-mCherry^NLS^* and *Dl^Gal4^ UAS-mCherry^NLS^*, *byn^Gal4^* and *Dl^Gal4^* were recombined with *P{UAS-mCherry^NLS^}.* For the construction of *byn^Gal4^ tubGal80^ts^*, *byn^Gal4^* was recombined with *P{tub-Gal80^ts^}*. For the construction of *byn^Gal4^UAS-ci^Rep^*, *byn^Gal4^* was recombined with *P{UAS-ci^76^}*. Stocks generated for temperature shift experiments were as follows: (i) *y w; P{tub-Gal80^ts^} ap^md544^/CyO, Dfd::YFP* [*ap^md544^* (*89*) is a strong *ap* hypomorph as well as a bona fide ap^Gal4^ driver; *ap^md544^/ap^DG3^* animals have almost no wings and halteres and die precociously]; (ii) *y w; P{UAS-mCD8::GFP} ap^DG3^ P{UAS-ap}/CyO, Dfd::YFP*; and (iii) *y w; P{UAS-mCD8::GFP} ap^DG3^/CyO, Dfd::YFP* (used as control).

### New *ap* alleles established for this study

#### Generation of the ap^MS1^ landing site

Preliminary observations suggested that a regulatory region required for adult survival is likely located in or between apCON1 and the proximal side of the apE wing enhancer (*51*). Therefore, this region was deleted and replaced with a landing site for the ɸC31 integrase system and a Flippase Recombination Target (FRT) using CRISPR-Cas9–mediated homology directed repair (HDR) (*90*). In preparation for this approach, two plasmids containing one guide RNA (gRNA) each were constructed in the following way. Plasmid pU6–Bbs I–chiRNA (*91*) was cut with the restriction enzyme Bbs I and ligated with the following oligonucleotides: gRNA “CRISPR L” located in apCON1, 5′-CTTCGTAAACTCAAGTATCGGAAT-3′ and 3′-ATTTGAGTTCATAGCCTTACAAA-5′; gRNA “CRISPR R” located in apCON2, 5′-CTTCGAAGCTGCACTATGACTGAG-3′ and 3′-CTTCGACGTGATACTGACTCCAAA-5′.

The newly generated plasmids are pMS393 (for CRISPR L) and pMS394 (for CRISPR R). Donor plasmid pMS398 was designed in silico and synthesized by Genewiz (Azenta Life Sciences). It consists of two ~700–base pair (bp) homology arms, which flank a cassette consisting of [55 bp attP]-[34 bp FRT]-[517 bp Dad13 enhancer]-[34 bp FRT]. The wing-specific Dad13 enhancer serves as selectable marker as described in (*46*).

Donor plasmid pMS398 (at 500 ng/μl) was injected together with the two gRNA-producing plasmids (pMS393 and pMS394; at 100 ng/μl) into embryos with genotype *y^1^ M{vas-Cas9}zh-2A w^1118^; +*. Successful HDR events lead to the deletion of 3143 bp from the *ap* locus and insertion of the “dominant marker” Dad13. Adult injectees were crossed with *y w* flies, and their progeny was screened for the weak dominant wing phenotype generated by the presence of the Dad13 enhancer in the *ap* locus (*46*). Candidates were verified by polymerase chain reaction (PCR) and sequencing, and a *y w; ap^MS1+Dad13^/CyO, Dfd::YFP* stock was established. In a second step, the Dad13 enhancer was removed by Flippase-mediated recombination (*92*). The new allele is called *ap^MS1^* (*51*). Homozygous flies show the precocious adult death phenotype but have normal wings. The sequences on the proximal and distal sides of this deletion are as follows: proximal side (attP underlined), TCCCGAACGAAAAGAAAAGAGGAGTAGTGCCCCAACTGGGGTAACCTTTGAGTTCTCTCAGTTGGGGGCGTAGGC; distal side (FRT underlined), GAAGTTCCTATTCTCTAGAAAGTATAGGAACTTCCGTAGTGTGCAATGCACACT.

#### *Generation of the* ap^MS2^ *landing site (*ap^ΔLSE^*)*

Although *ap^MS1^* flies are short-lived, complementation data suggested that not all of the LSE is missing on this chromosome (*51*). Therefore, the *ap^MS1^* deletion was extended on its distal side. This was achieved by Flippase-induced recombination between the FRTs present on *ap^MS1^* and *ap^R2^* (fig. S1F). This leads to a deletion of 3794 bp. The allele is called *ap^MS2^*. Note that it is referred to as *ap*^Δ*LSE*^ in the main text and in all figures (fig. S1D). The sequence on the distal side of this deletion is as follows (proximal side same as above): distal side (FRT underlined), GAAGTTCCTATTCTCTAGAAAGTATAGGAACTTCTATAATTTGACCGCAATTTT. As *ap^MS1^*, homozygous *ap^MS2^* flies show the precocious adult death phenotype, but they also lack wings and halteres because the apE wing enhancer is deleted as well (*44*, *45*).

#### *Construction of* ap^MS2+minLSE^ *(*ap^minLSE^*)*

The LSE (562-bp fragment) was cloned in reentry vector DB345 (*45*), and plasmid CR24 was obtained. It was injected into *y w vas-integrase; ap^MS2^/CyO* embryos. Successful integration into the *ap^MS2^* landing site was detected by the expression of the *yellow* reporter. The *y^+^* marker was subsequently removed by Flp-treatment (*92*). Flip-out candidates were verified by PCR and sequencing, and a balanced stock was established (fig. S1G).

#### *Construction of* ap^c1.4b-Gal4^ *(*ap^Gal4^*)*

*ap^c1.4b-Gal4^* was obtained by injection of plasmid pMS399 into *y w vas-integrase; ap^c1.4b^/CyO* embryos (fig. S1A). pMS399 contains a *mini-yellow* marker devoid of all *yellow* enhancers. Injectees were crossed with *y w* partners. Transgenic flies could be detected by their y^+^ wings. A *y w; ap^c1.4b-Gal4^/CyO* stock was established, and crucial parts of the insert were verified by sequencing. Note that reporter gene expression patterns generated by the classic *ap^md544^* allele (*90*) and *ap^c1.4b-Gal4^* are indistinguishable. However, unlike *ap^md544^*, hemizygous *ap^c1.4b-Gal4^* flies are well viable and fertile and have normal wings and halteres. Note that it is referred to as *ap^Gal4^* (fig. S1H) in the main text and in all figures.

The Gal4 gene in this and the following two *ap^Gal4^* alleles is under the control of a synthetic core promoter. It consists of a modified 155-bp fragment originating from the *evenskipped* gene and contains consensus motifs known to be present in potent *Drosophila* promoters. They are the TATA box, the initiator element, the motif ten element, and the downstream promoter element (*93*).

#### *Construction of* ap^DG1-Gal4^ *(*ap^ΔLSE-Gal4^*)*

*ap^DG1-Gal4^* was generated by Flp-mediated recombination between FRT sites present in *ap^c1.4b-Gal4^* and *PBac{RB}e01573* (*94*). The transgene insert *PBac{RB}e01573* marks the distal end point of the 27-kb intergenic spacer. The same strategy was previously used to generate *ap^DG1^* (fig. S1C) (*58*). Hence, all *ap* enhancers located in the intergenic spacer including the LSE are lost. With respect to survival, *ap^DG1-Gal4^* behaves like *ap*^Δ*LSE*^. Note that in *ap^DG1-Gal4^*, the *mini-yellow* marker of *ap^c1.4b-Gal4^* and the *mini-white* marker of *PBac{RB}e01573* have been deleted by the Flp-procedure. Also note that it is referred to as *ap*^Δ*LSE-Gal4*^ (fig. S1I) in the main text and in all figures.

#### *Construction of* ap^R1+LSE-Gal4^ *(*ap^LSE-Gal4^*)*

This Gal4 driver was obtained by insertion of plasmid pCR38 into the *ap^R1^* landing site (fig. S1E). In *ap^R1+LSE-Gal4^*, the whole 27-kb intergenic spacer is replaced by the 562-bp minimal LSE and the Gal4 gene. *ap^R1+LSE-Gal4^* flies are viable and fertile, indicating that the LSE is complementing for the deleted intergenic spacer. This suggests that the LSE also activates the Gal4 gene accordingly. Note that the *yellow* marker present on pCR38 remains in place and is located between the 3′ end of Gal4 and the 5′ end of *l(2)09851*. This *yellow* marker contains the body color enhancer but not the wing enhancer of the *yellow* gene. Note that *ap^R1+LSE-Gal4^* is referred to as *ap^LSE-Gal4^* (fig. S1J) in the main text and in all figures.

#### *Construction of* ap^MS3-Gal4^ *(*ap^LSE-Gal4_2^*)*

*ap^MS3-Gal4^* was obtained by Flp-mediated recombination between the FRT sites in *ap^c1.4b-Gal4^* and *ap*^*MS1*Δ*Dad13*^ located within the *ap^LSE^* enhancer. Note that the *mini-yellow* marker of *ap^c1.4b-Gal4^* is lost and that the distal break points of *ap^MS1^* and *ap^MS3-Gal4^* are identical. Hence, the apE wing enhancer is still in place. Also note that it is referred to as *ap*^Δ*LSE-Gal4_2*^ (fig. S1K) in the main text and in all figures.

### Sample preparation and immunohistochemistry

#### G-TRACE

To follow *ap* expression throughout different stages of development, Gal4 driver lines were crossed with the real-time and clonal expression stock [G-TRACE; (*61*)]. Dechorionated embryos or dissected tissue samples were fixed with 4% paraformaldehyde in phosphate-buffered saline (PBS) for 25 min at room temperature (RT). Samples were washed three times for 20 min each with PBS at RT and finally mounted in Vectashield containing 4′,6-diamidino-2-phenylindole (DAPI) (Vector Laboratories). Confocal imaging was performed using a Leica SP5 microscope or an Olympus SpinD. Image processing was done with the ImageJ software.

#### Immunostaining of embryonic hindguts

To verify the specificity of the Ap expression generated by G-TRACE stainings, antibody stainings against Ap was performed. Standard immune-detection protocols were followed to image late-stage embryos. Primary antibody used in this study was rabbit α-Ap [1:800 to 1000; (*45*)]. Secondary antibody was α-rabbit Alexa Fluor 488 used at 1:500. Samples were mounted in Vectashield containing DAPI. Confocal imaging was performed using a Leica SP5 microscope or an Olympus SpinD. Image processing was done with the Omero or with the ImageJ software.

#### Immunostaining of adult brains

Brains from 1-day-old adult females were dissected in ice cold PBS, fixed in 4% paraformaldehyde in PBST (PBS containing 0.3% Triton X-100) for 20 min at RT, washed three times for 20 min in PBST, and blocked in PBST containing 5% normal goat serum (NGS) overnight at 4°C. Samples were incubated with primary antibodies in PBST containing 5% NGS for 48 hours at 4°C (mouse α-Brp 1:100, Developmental Studies Hybridoma Bank (DSHB) AB_2314866; chicken α-GFP 1:1000, Abcam ab13970; rat α-Ilp2 1:100, obtained from P. Léopold, Institute Curie, Paris, France). Samples were washed three times for 20 min in PBST and incubated with respective secondary antibodies (1:1000) in PBST containing 5% NGS for 48 hours at 4°C (goat α-mouse Alexa Fluor 633 nm, Invitrogen A21050; goat α-chicken Alexa Fluor 488 nm, Invitrogen A11039; goat α-rat Alexa Fluor Plus 488 nm, Invitrogen A48262). Samples were washed three times for 20 min in PBST and mounted in Vectashield (Vector Laboratories).

#### Image acquisition and quantification of adult brains

Samples were imaged on a Zeiss LSM700 point scanning confocal microscope using a 20×/0.8 numerical aperture objective (air). *Z* stacks containing the entire volume of the brains or the regions of interest were acquired. For quantification of fluorescence intensity in the IPCs of the brain, the same confocal settings were applied for both experimental groups, and sum projection images of *z* stacks in grayscale were analyzed with the ImageJ software. The freehand tool was used to draw IPC clusters, and the mean pixel intensity was calculated after subtracting the background intensity.

#### Ampulla preparations

Adult ampullae were dissected in 1× PBS on a siliconized slide and mounted in 1× PBS*/*50% glycerol. To prevent crushing of the tissue samples, coverslips with tiny playdough feet were used. Preparations were immediately inspected with a Leica DM2700M microscope equipped with Normarski optics and imaged with a Leica Flexacam C3 camera.

### Micro–computed tomography

μ-CT was used as a noninvasive imaging method to achieve high-resolution visualization of the pupal and adult hindgut. μ-CT scans were done as previously described (*72*, *73*). In short, female pupae were staged and pooled in Eppendorf tubes containing 1 ml of 0.5% Triton X-100 (Sigma-Aldrich, T8787) in PBS, incubated at 100°C for 20 s in a heatblock, and then allowed to cool down at RT for 5 min. This kills the pupae immediately so that they do not progress to later developmental stages and partially permeabilizes the cuticle. One-day-old female virgins were anesthetized with CO_2_. Five to 50 flies were pooled in a 1.5-ml Eppendorf tube. One milliliter of 0.5% Triton X-100 in PBS (PBST) was then added for around 5 min or until flies sunk to the bottom of the tube. For both stages, PBST was exchanged for 1 ml of Bouin’s solution (Sigma-Aldrich, HT10132) for 16 to 24 hours to fix the samples, which were then washed three times for 30 min and then again overnight with PBST. They were stained in 1 ml of 1:1 Lugol’s solution (Sigma-Aldrich, 62650) in ultrapure H_2_O for a minimum of 3 days. To remove the staining solution, the samples were washed with ultrapure water once and then stored at RT in ultrapure water until ready to scan. Samples were finally mounted head down in a 10-μl pipette tip filled with ultrapure water, and the ends were sealed with parafilm. Five to 10 flies were mounted per tip.

Pupae and flies were imaged on a Bruker Skyscan 1272 with the following settings: 40 kV, 110 μA, 4 W, CMOS (complementary metal-oxide semiconductor) camera scanning at a 2.95-μm pixel size, 0.3 to 0.35 rotation step, 30-μm random movement, and four frame averaging. Images were reconstructed using the Bruker NRecon software, and images were further processed by background subtraction and Gaussian smoothing in ImageJ (FIJI v2.14.0/1.54f).

The software ITK-snap v3.8.0 was used to manually segment each intestine with the polygon tool. Organ perimeters were segmented every 15 to 20 slices in the axial plane, and the morphological interpolation tool was used to fill in the spaces. Smoothing factors were added when needed. Manual corrections were performed using the adaptive paintbrush tool. For 3D visualization, segmentations were converted into meshes and were further processed with the software Meshlab. Meshes were processed with the quadric edge collapse decimation to reduce the number of faces to 10% and/or smoothened using HC Laplacian smoothing.

### Survival assays

#### Quantification of survival rates

Unless otherwise stated, survival rates for the various genotypes were carefully scored over a period of 18 days. Flies of both sexes were collected during 1 day and kept at 25°C in pools of 20 to 30 flies. Flies were kept in glass vials (~56 cm^3^) on a normal cornmeal agar diet not supplemented with fresh baker’s yeast. Flies were transferred to fresh tubes at least every second day without CO_2_ anesthesia. The number of surviving flies was scored every third day. Survival curves are plotted as survival in percent [(survivors on day X)/(total of flies scored) × 100] versus day X after eclosion.

#### Survival under high-salt conditions

As above but standard food was supplemented to reach a final concentration of 250 mM NaCl.

#### *Survival of starved* y w *flies*

Freshly eclosed *y w* females and males were collected separately and directly transferred to starvation tubes (1.5% agar in water) or vials containing standard food. Flies kept on normal food were aged for 5 days before they were transferred to starvation tubes. Survival was scored until the last fly died.

#### *Survival of butt-plugged* y w *flies*

Butt-plugged flies were produced by sealing the anus of freshly eclosed *y w* females and males with a drop of superglue (Pattex, no. 2804584) under CO_2_ anesthesia. Thereafter, females and males were kept in separate tubes containing standard diet. Survival was scored until the last fly died. During this period, flies were transferred into fresh tubes at the latest every second day.

### Feeding, excretion, and PE assays

#### flyPAD

To test if the *ap* mutant flies are interacting with food or not, flyPAD assays were performed (*34*, *95*). Food pellet composition was as follows: 5% (w/v) sucrose (granulated sugar, Tate & Lyle), 10% (w/v) Brewer’s yeast (no. 903312, MP Biomedicals), and 1.5% (w/v) agar (Sigma-Aldrich, A7002) supplemented with Nipagin [Sigma-Aldrich, H5501; 30 ml/liter of 10% (w/v) Nipagin in 95% EtOH]. Nipagin was added once the food had cooled down to <60°C. The food was poured in 5-cm petri dishes. Solid food pellets were punched out using a P1000 cut tip to match the exact diameter of the central electrode patch.

One-day-old virgins were then individually transferred to the flyPAD arena by mouth aspiration. Half of the wells were filled with a food pellet, and the other one was left empty. Virgins were allowed to feed for 1.5 hours at 25°C. Only the last 60 min were used for quantification. flyPAD assays were only carried out during the feeding peak between 10 a.m. and 2 p.m. The total number of sips per fly was acquired with the Bonsai framework and further analyzed in MATLAB.

#### Blue food squash assay

The amount of ingested food was quantified with food containing 1% Brilliant Blue FCF (*34*). In short, virgins were collected over 1 day. Later in the afternoon, they were transferred to starvation tubes (1.5% agar in water). On the following day, after around 16 hours of starvation, the flies were transferred into tubes containing blue food and allowed to feed for a maximum of 20 min. They were subsequently frozen in liquid nitrogen and transferred in groups of three to a clean 2-ml PCR tube (Eppendorf, 22431048) with 0.5 ml of water and a 5-mm stainless-steel metal bead (QIAGEN, 69989). The flies were then homogenized using a QIAGEN TissueLyser II for 90 s at 30 Hz. The samples were centrifuged at 10,000*g* for 5 to 10 min. Two hundred microliters of the supernatant per sample was directly transferred into individual wells of a 96-well, flat-bottom, optically clear plate (Thermo Fisher Scientific, Sterilin, 611F96). A BMG Labtech FLUOstar Omega plate reader was used to measure dye content by reading the absorbance at 629 nm. We used a standard curve of pure FCF blue dye to calculate the dye content ingested per fly.

#### Crop-mount assay

Flies were prepared the same way as described for the blue food squash assay. Instead of homogenizing them, their foregut and crop were quickly dissected and mounted on a poly-lysine–coated slide. With a normal bright-field microscope (Leica DM2700M) attached to a camera (Leica Flexacam C3), the crops were imaged. Furthermore, their area was manually analyzed using the ImageJ software.

#### FlyPoo assay

Virgins were prepared the same way as described for the blue food squash assay. Instead of putting them into vials containing blue food, 30 flies were transferred into individual petri dishes (Sarstedt, no. 82.1194.500) containing a wedge of blue diet. They were allowed to eat and poop for 2 hours. Afterward, the flies were removed, and the excreted feces on the petri dishes were manually counted.

#### Meconium excretion assay

To characterize the rate and dynamics of meconium excretion, the following assay was performed: Freshly eclosed females and males (maximum of 15 min old) were collected with the shortest possible CO_2_ anesthesia. Around 15 to 25 flies for each sex were transferred into petri dishes (Sarstedt, no. 82.1194.500) containing a wedge of standard food or into an empty petri dish without any food. Every time point relied on a different cohort of flies to ensure a real-time snapshot of meconium excretion. Flies were immediately removed after the desired time period so that only material produced during a certain interval was counted. The total number of excreted feces was manually counted for each sex, petri dish, and time point. The surface of the petri dish was inspected under a light microscope for this purpose. Scoring was performed directly after removal of the flies, and all feces on the surface, the wall, and the lid of the petri dish were counted. The total number of excreta was then divided by the number of flies present in each petri dish. The experiments were not performed and quantified in a blinded manner with respect to the fly genotypes.

#### Adult initial feeding assay

To analyze when juvenile flies start to eat after eclosion, the adult initial feeding assay was performed. Flies were prepared the same way as for the meconium excretion assay. Instead of petri dishes supplemented with standard food, 10 to 20 flies were transferred into a petri dish (Sarstedt, no. 82.1194.500) containing a wedge of blue diet (same composition as for the blue food squash assay). Every hour, the first appearance of blue food in the fly’s intestine was scored by eye. For the time points 10 and 24 hours after eclosion, new batches of flies were used. The number of excreted meconium as well as the number of excreted blue diet was manually counted.

#### PE assays

For video-assisted analysis of the various genotypes, virgins were collected over 1 day and kept in vials filled with the same food as described in the flyPAD section until their behavior was recorded on the following morning from ZT0.5 to ZT1.5 or ZT1.5 to ZT2.5, or on the following evening from ZT12 to ZT13 (four individual flies per 1-hour interval). For the morning experiments, 1-day-old flies were loaded into the arenas on the same day 15 min prior to the experiment. For the evening experiments, 1-day-old flies were loaded into the arenas at ZT0-1 and analyzed at ZT12-13 on the same day. During the recording, flies have unrestricted access to normal food (for food content, see flyPAD assay). For experiments described in Fig. 3 (D and E), starved and well-fed *ctrl* flies were put on starvation food (1.5% agar). The behavior experiments were done using a custom-built 3D-printed chamber comprising four individual small chambers as described previously (*96*, *97*). In brief, flies were placed in the chamber and recorded for a period of 1 hour with an aCA2440–75 μm camera (Basler) mounted with a 0.5× 2/3” Telecentric lens (GoldTL, Edmund Optics) equipped with a near-infrared bandpass filter (Midopt BP850-58). Illumination was achieved by directing the light from the infrared light-emitting diode (M850L3, Thorlabs) onto a glass surface positioned at a 45° angle above the chamber, enabling reflection of the light downward. The flies were recorded at a resolution of 142 pixels/mm (1386 pixels by 1066 pixels). Manual annotation of the PE was conducted using Behavioral Observation Research Interactive Software [BORIS; (*98*)]. Three types of PE were identified: PE on food, PE while moving, and PE while immobile. The start of a PE event was considered as the first frame where labellum extension was observed, and the end was defined as the frame after the proboscis was completely retracted. For the immobility analysis, videos were analyzed using SLEAP (*99*) to track the head, thorax, and abdomen tip. The fly was considered immobile when there was no movement of all three points. This method excludes periods of grooming, as either the head or the abdomen moves during this behavior. A fly is considered to enter a sleep period if it remains immobile for more than 5 min. Data were analyzed using an in-house Python script developed for this study.

### Temperature shift experiments

#### *Determining the temporal requirement for* ap *expression with respect to the precocious adult death phenotype*

To address the temporal requirement for *ap* expression with respect to the adult precocious death phenotype, the following experimental procedure was used. *y w; ap^md544^ tub-Gal80^ts^/CyO, Dfd::YFP* virgins were crossed with *y w; UAS-mCD8::GFP ap^DG3^ UAS-ap/CyO, Dfd::YFP* males. Embryos were collected in a fresh culture tube containing standard cornmeal agar supplemented with fresh baker’s yeast at 18°C for 24 hours. Parents were removed, and the collected animals were further aged at 18°C for 96, 120, 144, 168, 192, 216, 240, 264, 288, 312, or 336 hours before cultures were shifted to 29°C and kept at this temperature until adulthood. Then, the survival of emerging adults with the genotype *UAS-CD8::GFP ap^DG3^ UAS-ap/ap^md544^ tub-Gal80^ts^* was carefully scored.

*UAS-mCD8::GFP ap^DG3^ UAS-ap/ap^md544^ tub-Gal80^ts^* animals continuously grown at 18°C did not show a GFP pattern in third instar larvae. Furthermore, adults did not form wings and showed the precocious adult death phenotype. At 29°C constantly, an *ap*-like GFP pattern was seen in third instar larvae, indicating that Gal80^ts^ is no longer functional and that Gal4 activates the UAS-transgenes. Consequently, adults developed normal wings and survived well (table S3).

To induce transient *ap* expression in the hindgut, *ap^md544^* tub-Gal80^ts^*/CyO* flies were crossed with *y w; UAS-mCD8::GFP ap^DG3^ UAS-ap/CyO, Dfd::YFP* or *y w; UAS-mCD8::GFP ap^DG3^/CyO, Dfd::YFP* (as negative control) flies. Embryos were collected at 18°C for 24 hours. Then, parents were removed. The cultures were aged at 18°C for 216 hours. To induce the Ap pulse, cultures were shifted to 29°C for 48 hours. Afterward, animals were shifted back to 18°C until eclosion. Adults of the correct genotype could be easily detected because of their strong wing phenotype. For the behavioral assays, the flies were prepared as described above.

#### *Determining the temporal requirement for the* byn^Gal4^ > ci^Rep^ *effect on papillae development*

*byn^Gal4^ tub-Gal80^ts^/TM6C* virgins were crossed with homozygous *y w; UAS-ci^Rep^* males. When *byn^Gal4^ tub-Gal80^ts^/UAS-ci^Rep^* animals were constantly grown at 18°C, adults survived well. Dissected female ampullae contained four papillae. When constantly grown at 29°C, adults also survived well, but they had no papillae (table S4). For the temperature shifts, the experimental procedure was as follows. The same cross as above was set up. Embryos were collected in a fresh culture tube containing standard cornmeal agar supplemented with fresh baker’s yeast at 18°C for 24 hours. Parents were removed, and the collected animals were aged at 18°C for 48, 96, 144, 192, 240, 264, or 288 hours before cultures were shifted to 29°C. Cultures were then kept at this temperature until adulthood. Once adults started to emerge, the phenotypic classes were scored (Sb versus non-Sb flies). In addition, the survival and fertility of non-Sb flies (*byn^Gal4^ tub-Gal80^ts^/UAS-ci^Rep^*) was scored, and the hindguts of several females were inspected for the presence or absence of rectal papillae.

### Locomotion assays

For the locomotion assays, virgin *y w* flies were collected 1 day before the experiment (juvenile group) or 5 days before the experiments. *ap*^Δ*LSE*^ flies were collected 1 day prior to the experiment as they tend to die within the first few days. For the starved condition, *ctrl* flies were kept for 1 day on agar medium. Flies were placed in 65-mm vials containing either standard food or agar medium (for the starved condition). We used *Drosophila* ARousal Tracking system [DART; (*97*, *100*)] to track the positions of the flies for ~24 hours. Videos were recorded at 5 fps and analyzed using DART software. We compared the total distance traveled between groups for the same number of hours.

### Statistical analysis

Imaging and behavioral data were analyzed in GraphPad Prism v10 with the exception of the PE and behavioral state ratios, which were analyzed in Python. For PE and behavioral state ratios, group means were compared across groups using Permutational Multivariate Analysis of Variance (PERMANOVA) (9999 permutations). Category-specific differences were assessed by applying centered log-ratio (CLR) transformation followed by Analysis of Variance (ANOVA) and Tukey’s post hoc test on the CLR-transformed components with Holm correction. See tables S1 and S2 for statistical details.
