# Supplementary material for: Intestinal obstruction impairs feeding and promotes sleep in Drosophila melanogaster
Source: Sci Adv. 2026 May 20;12(21):eady2183. doi: 10.1126/sciadv.ady2183 (PMC13189133; doi:10.1126/sciadv.ady2183)
Supplement: Supplementary file 1 — Figs. S1 to S19 Tables S1 to S4 Legends for movies S1 to S4 [file sciadv.ady2183_sm.pdf]

Science Advances
AAAS

Supplementary Materials for

Intestinal obstruction impairs feeding and promotes sleep in *Drosophila melanogaster*

Cindy Reinger *et al.*

Corresponding author: Martin Müller, m.mueller@unibas.ch; Anissa Kempf, anissa.kempf@unibas.ch

Sci. Adv. **12**, eady2183 (2026)
DOI: 10.1126/sciadv.ady2183

The PDF file includes:

Figs. S1 to S19
Tables S1 to S4
Legends for movies S1 to S4

Other Supplementary Material for this manuscript includes the following:

Movies S1 to S4

Figure S1.

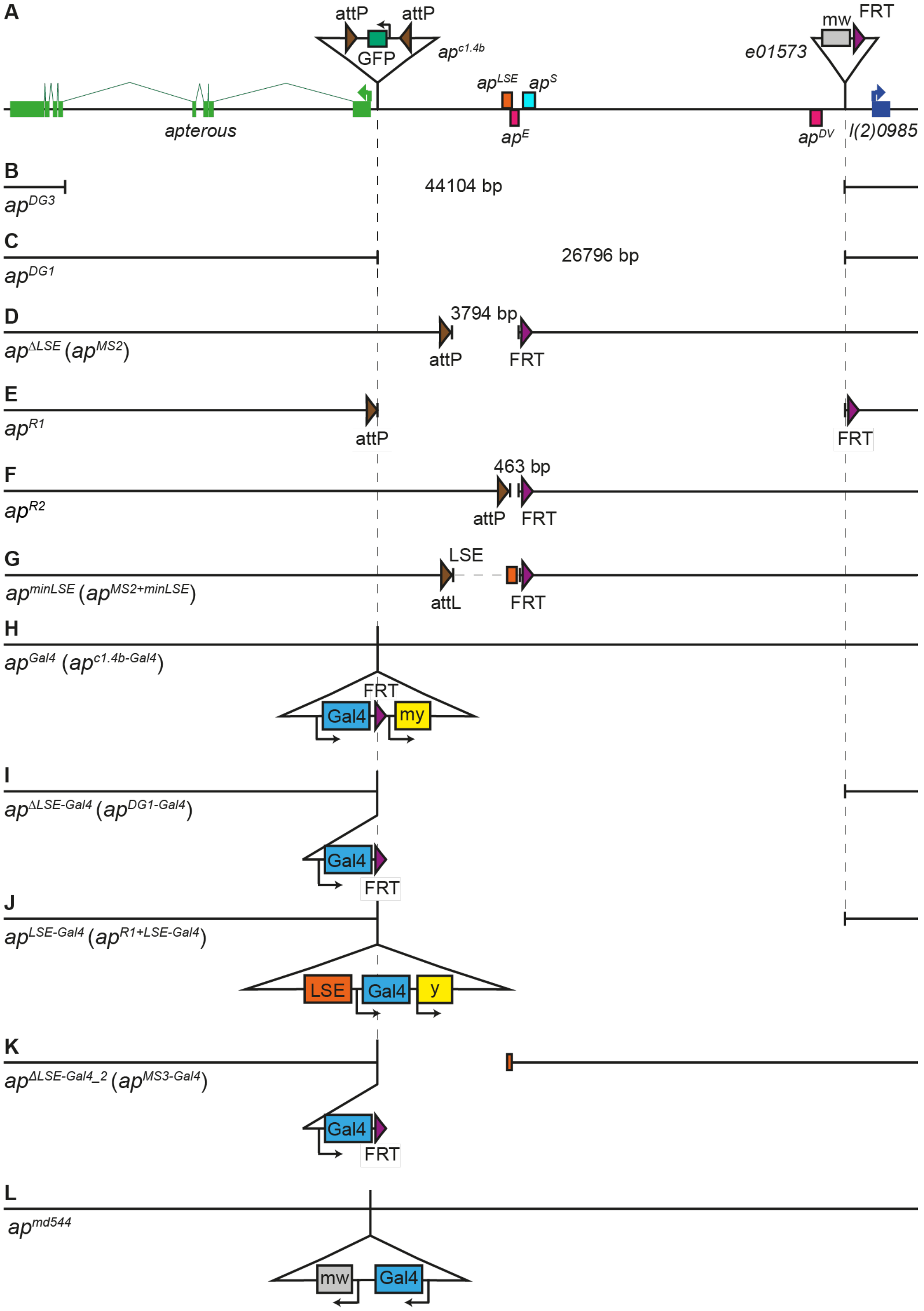

Figure S1. Overview of fly lines used for experiments. Note that only genetic elements relevant for this study are shown. **A.** The *apterous* (*ap*) locus is depicted in green and is flanked by *l(2)09851* (in blue) on its distal side. Four enhancers mentioned in the text are sitting within the intergenic spacer: the lifespan enhancer (ap^{LSE}) (orange), the wing-related enhancers ap^{E} and ap^{DV} (pink), and the central nervous system (CNS) enhancer *apS* (turquoise blue). *apS* maps to a ~2.4 kb interval to the right of *apE* (*60*). Insert $ap^{c1.4b}$ is located 400 bp upstream of the transcription start site (*44*). It contains a *GFP* reporter (dark green) flanked by two attP sites (brown), which can be used for recombination-mediated cassette exchange (*101*). *P{BacRB}e01573* (ap^{e01573}; (*94*)) contains the *mini-white* (*mw*) marker and an FRT site. It marks the distal end point of the 27 kb intergenic spacer. **B.** In ap^{DG3}, a large part of the *ap* locus including most enhancers including the LSE is deleted. Proteomics data indicates that ap^{DG3} is a *bona fide* null allele (*51*).

(**Figure S1 continued**). **C.** In ap^{DG1}, the intergenic spacer including the LSE is deleted (*58*). **D.** In $ap^{\Delta LSE}$ (also ap^{MS2}), 3794 bp are deleted including the LSE and apE. This DNA is replaced by an attP and FRT site. **E.** Like ap^{DG1}, ap^{R1} lacks the 27 kb intergenic spacer, but it contains strategically positioned attP and FRT recombination sites. **F.** ap^{R2} was used to generate $ap^{\Delta LSE}$ through Flp-mediated recombination between the FRTs in ap^{MS1} and ap^{R2} (85). In ap^{R2}, the 463 bp apE wing enhancer is deleted (*46*). **G.** The LSE (562 bp fragment) was cloned in the reentry vector DB345 and the transgenic fly line $ap^{MS2+minLSE}$ (ap^{minLSE}) was established (*51*). The *mini-yellow* marker was deleted by Flp-mediated recombination. Consequently, only attL remains in place and attR is deleted. **H.** ap^{Gal4} (also $ap^{c1.4b\text{-}Gal4}$) was obtained by injecting plasmid MS544 into a fly stock containing the $ap^{c1.4b}$ landing site (Fig. S1A). It further contains a FRT and a *mini-yellow* (*my*) marker. **I.** $ap^{\Delta LSE\text{-}Gal4}$ (also $ap^{DG1\text{-}Gal4}$) was generated by Flp-mediated recombination between FRT sites present in $ap^{c1.4b\text{-}Gal4}$ and ap^{e01573}. **J.** $ap^{LSE\text{-}Gal4}$ (also $ap^{R1+LSE\text{-}Gal4}$) was obtained upon insertion of plasmid CR38 into the landing site ap^{R1}. The entire 27 kb intergenic spacer is replaced by the minimal LSE and the Gal4 gene. Note that the *yellow* marker on CR38 remains in place and is located between the 3' end of Gal4 and the 5' end of *l(2)09851*. **K.** $ap^{\Delta LSE\text{-}Gal4\_2}$ (also $ap^{MS3\text{-}Gal4}$) was obtained by Flp-mediated recombination between FRT sites in $ap^{c1.4b\text{-}Gal4}$ and ap^{MS1}. Note that a small part of the LSE remains in place (*51*) and that the *mini-yellow* marker of $ap^{c1.4b\text{-}Gal4}$ is lost. **L.** ap^{md544} is the classical Gal4 enhancer-trap of Calleja et al (*89*). Due to its insertion site just next to the transcription start site, it is also a strong *ap* allele. It is marked with *mini-white (mw)*.

Figure S2.

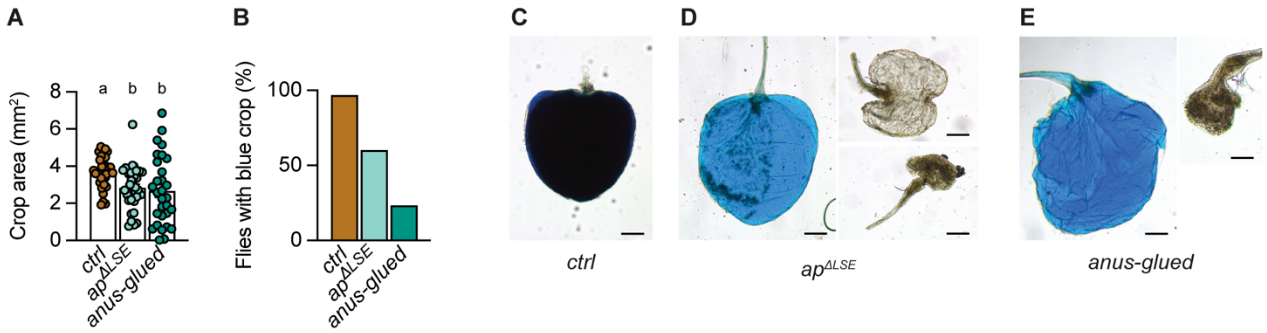

Figure S2. Crop function is not disrupted in constipated flies. A. The crop is a unique foregut organ in dipteran flies that affects many physiological and behavioral functions. Its function relies on an elaborate sphincter and pump system that moves stored nutrients to the crop, oral cavity, and the midgut. In *Drosophila*, all food transits through the crop and crop size can vary in response to signals obtained from innervating neurons. Well-fed flies often have a greatly enlarged crop (*34*). Since *ap*$^{\Delta LSE}$ mutants barely eat, we reasoned that this might be due to a dysfunctional crop. Therefore, crop size of *ap*$^{\Delta LSE}$ mutants were measured as previously described (*34*). Although the crop size area is on average significantly reduced in constipated mutant flies, some crops are able to expand (see also Fig. 2F, bottom). Therefore, reduced crop expansion in *ap*$^{\Delta LSE}$ flies is a consequence of altered food ingestion and not of impaired crop function. Similar observations were made for *ctrl* flies with a sealed anus. Genotype effect: $P = 0.0015$, Kruskal Wallis ANOVA, n = 30-34. Data are means ± s.e.m. **B.** Nearly 100% of *ctrl* flies have crops containing blue food after being starved and subsequently refed with blue food as opposed to *ap*$^{\Delta LSE}$ mutants and *ctrl* flies with a sealed anus. n = 30-34. **C-E.** Representative images of crops dissected from one-day-old *ctrl* (**C**), *ap*$^{\Delta LSE}$ (**D**) and sealed anus (**E**) flies after overnight starvation and a 20 min refeed period. Crops of *ctrl* flies are fully expanded and filled with blue food. Crops of sealed anus and *ap*$^{\Delta LSE}$ flies are either expanded and contain small amounts of blue food, or they are not enlarged and empty. Virgin female flies were used for all experiments. *n*: number of flies. Groups that do not differ significantly share the same letter, while groups with different letters are statistically significant. For statistical details see Table S1 and for detailed genotype descriptions see Table S2.

Figure S3.

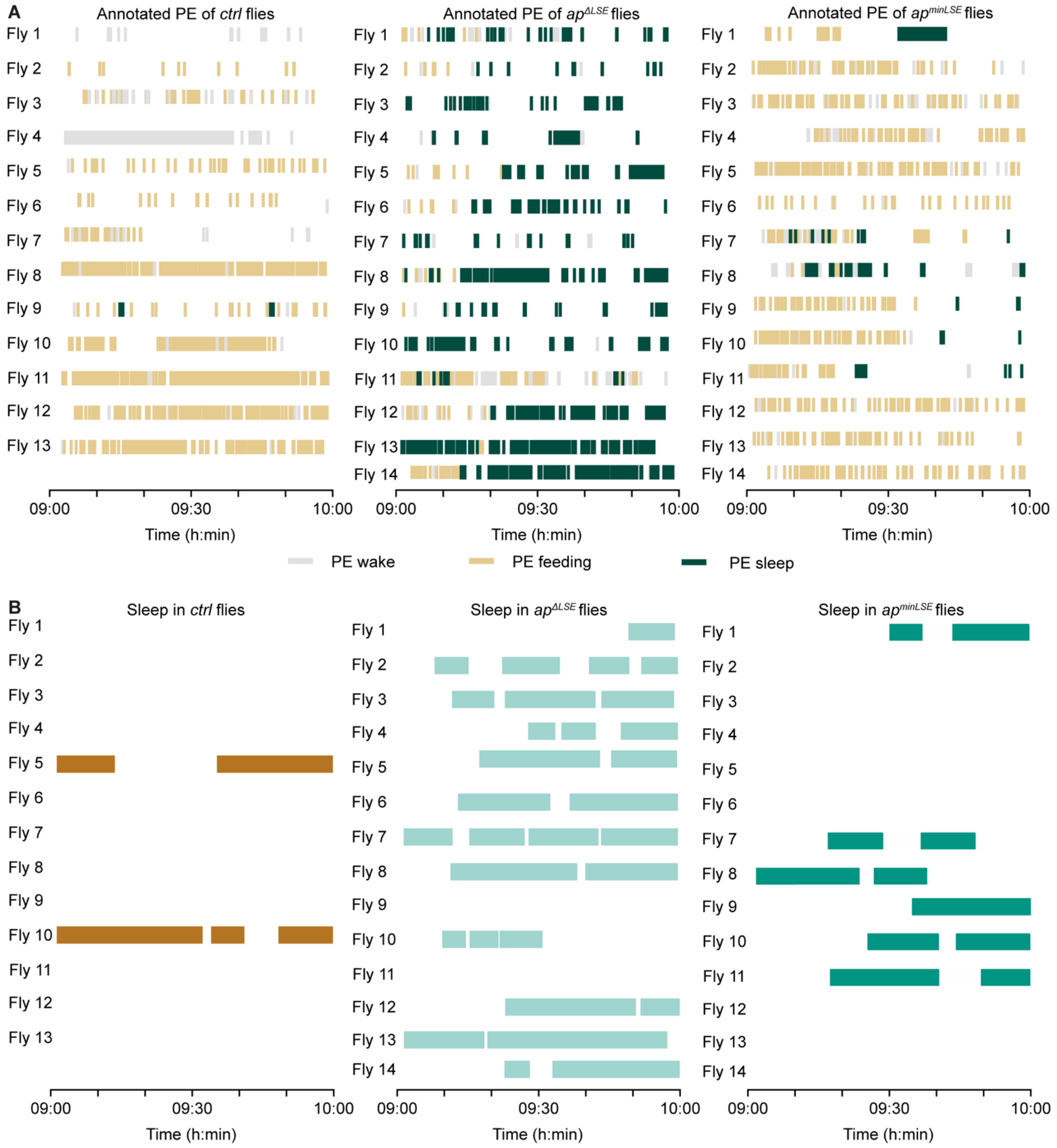

Figure S3. Rasterplots of proboscis extension (PE) events and sleep bouts in *ctrl*, *ap*$^{\Delta LSE}$ and *ap*^{minLSE} flies. A. Rasterplots of manually annotated PE events for individual *ctrl* (left)**,** *ap*$^{\Delta LSE}$ (middle) and *ap*^{minLSE} (left) flies are shown (the color code for PE wake (while active), PE feeding (towards food) and PE sleep (during sleep) is shown below the plots). *ctrl* and *ap*^{minLSE} flies tend to extend their proboscis toward food throughout the entire time of recording. *ap*$^{\Delta LSE}$ flies tend to extend their proboscis toward food or while active at the beginning of the recording and then mainly display PE during sleep. **B.** Rasterplot of sleep bouts lasting at least 5 min for *ctrl* (left), *ap*$^{\Delta LSE}$ (middle) or *ap*^{minLSE} (right) flies. Virgin female flies were used for all experiments. See Fig. 2A-C for quantification.

Figure S4.

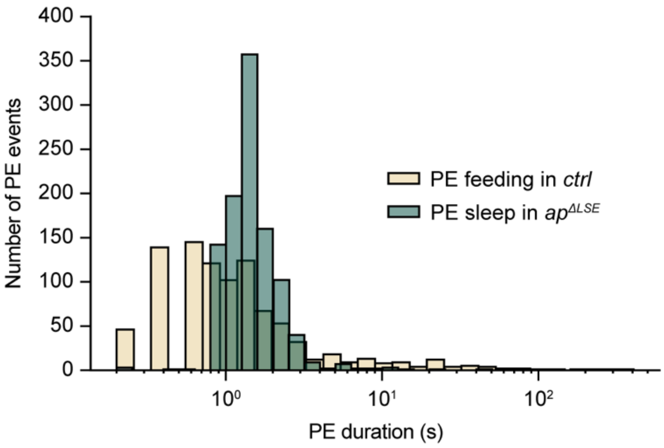

Figure S4. Dynamics of proboscis extension (PE) events. Comparison of the PE dynamics of the duration of individual PE toward food (yellow) or during sleep (green). PE toward food has more variability (duration ranges from less than 1 to 10 sec) while the duration of PE during sleep is more constant at around ~1-1.5 sec. The x-axis scale is logarithmic. Virgin female flies were used for all experiments.

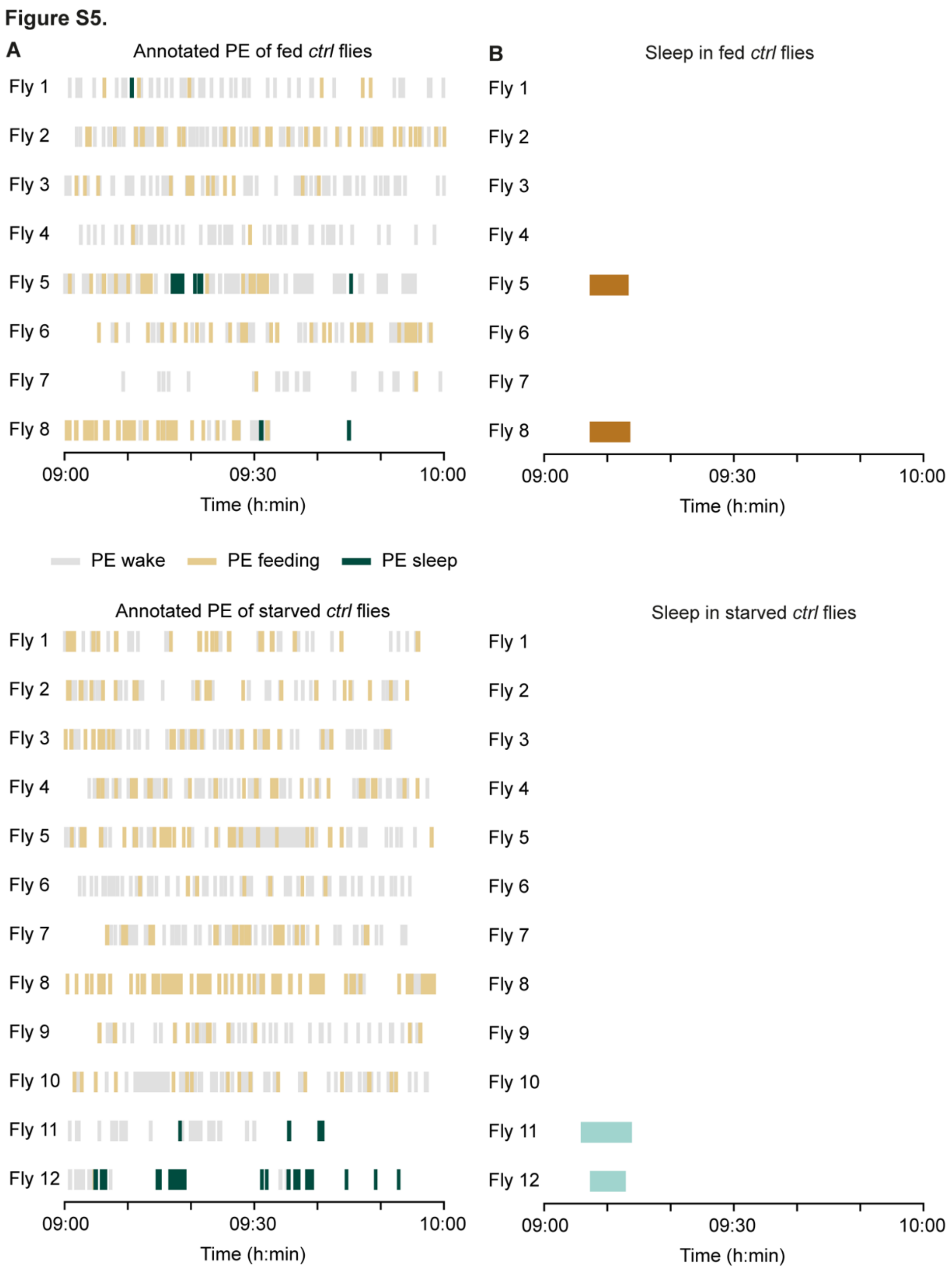

Figure S5. Rasterplots of proboscis extension (PE) events and sleep bouts in fed and starved flies. A. Rasterplots of manually annotated PE events for fed (top) and starved (bottom) *ctrl* flies, which were kept on starvation food during the experiment, are shown (the color code for PE wake (while active), PE feeding (towards food) and PE sleep (during sleep) is shown below the plots). Fed and starved flies did not behave differently during the 1 h recording period and perform mostly PE wake and PE feeding events. **B.** Rasterplot of sleep bouts lasting at least 5 min for fed (top) and starved (bottom) *ctrl* flies. Both fed and starved flies rarely sleep. Virgin female flies were used for all experiments. See Fig. 2D-E for quantification.

Figure S6.

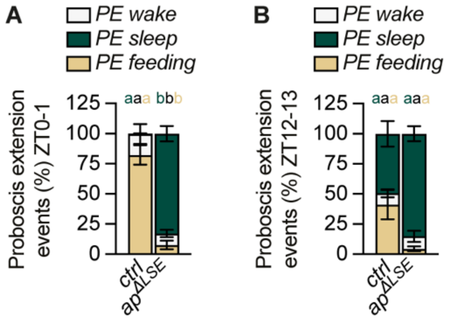

Figure S6. Comparison of PE events of *ctrl* and *ap*$^{\Delta LSE}$ flies during day- versus nighttime. A. During daytime (ZT0-1), *ctrl* flies direct most of their PEs towards food. In contrast, *ap*$^{\Delta LSE}$ flies show mostly PE sleep events. (genotype effect: $P < 0.0001$, PERMANOVA, n = 13-14). **B.** Annotated PE ratios show that *ctrl* flies increase their PE during sleep at nighttime (ZT12-13) and that the *ap*$^{\Delta LSE}$ flies behave similarly during day- and nighttime (genotype effect: $P = 0.03$, PERMANOVA, n = 10). Virgin female flies were used for all experiments. Data are means ± s.e.m. n: number of flies. Groups that do not differ significantly share the same letter, while groups with different letters are statistically significant. For statistical details see Table S1 and for detailed genotype descriptions see Table S2.

Figure S7.

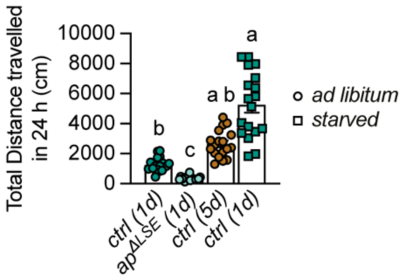

Figure S7. Locomotion analysis of *ap*$^{\Delta LSE}$ and *ctrl* flies under *ad libitum* or starvation conditions over a period of 24 h. *ap*$^{\Delta LSE}$ flies exhibit significantly reduced locomotor activity compared to fed or starved one-day-old control flies, as well as 5-days-old control flies. Starvation for 24 h prior to and during the recording did not increase locomotor activity in *ap*$^{\Delta LSE}$ flies. Genotype effect: $P < 0.0001$, Kruskal Wallis ANOVA, n = 17-18. Virgin female flies were used for all experiments. Data are means ± s.e.m. n: number of flies. Groups that do not differ significantly share the same letter, while groups with different letters are statistically significant. For statistical details see Table S1 and for detailed genotype descriptions see Table S2.

Figure S8.

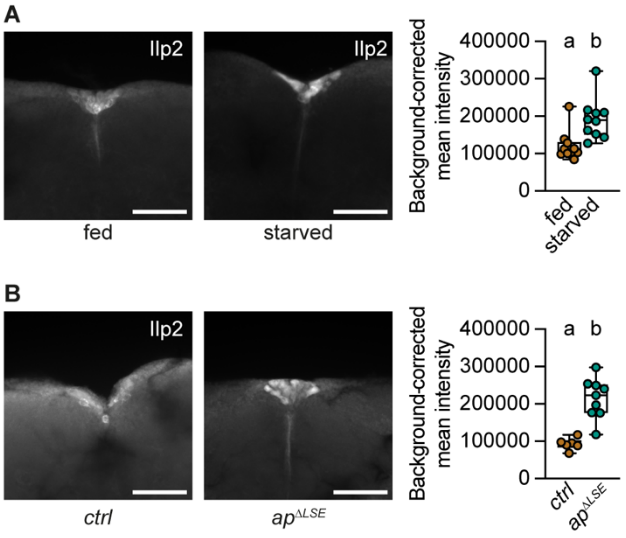

Figure S8. Assessment of Ilp2 expression in *ap*$^{\Delta LSE}$ flies. A. Representative images of Ilp2 immunostaining in the brains of one-day-old control flies maintained under fed or starved conditions (left). Quantification of background-subtracted mean fluorescence intensity reveals a significant increase in Ilp2 signal in starved control flies (right) (n = 10). P = 0.0021, Mann Whitney test. **B.** Representative images of Ilp2 immunostaining in the brains of one-day-old control and *ap*$^{\Delta LSE}$ flies maintained under ad libitum conditions (left). Quantification of background-subtracted mean fluorescence intensity shows a significant increase in Ilp2 signal in *ap*$^{\Delta LSE}$ flies compared to controls (n = 6-9). P = 0.0004, Mann Whitney test. Virgin female flies were used for all experiments. Scale bar: 50 µm. n: number of flies. Groups that do not differ significantly share the same letter, while groups with different letters are statistically significant. For statistical details see Table S1 and for detailed genotype descriptions see Table S2.

Figure S9.

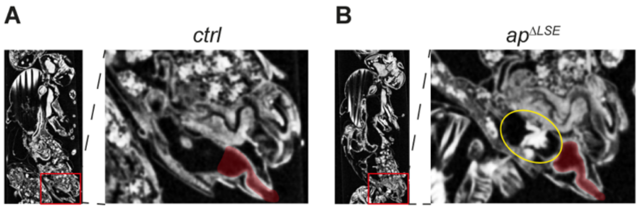

Figure S9. The anus of *ap*$^{\Delta LSE}$ flies is intact. A. μ-CT scan of a one-day-old *ctrl* fly. **B.** μ-CT scan of a one-day-old *ap*$^{\Delta LSE}$ fly. Red-colored areas indicate that anus is intact and not blocked. The Reinger's knot in *ap*$^{\Delta LSE}$ is highlighted with a yellow circle. Virgin female flies were used for all experiments.

Figure S10.

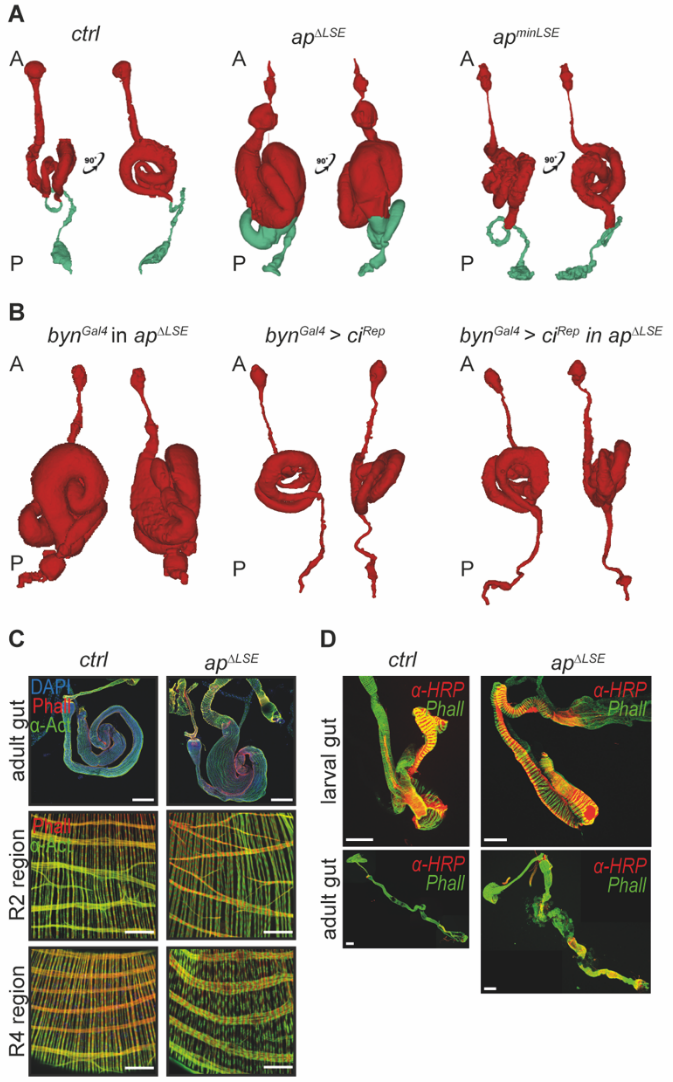

Figure S10. $ap^{\Delta LSE}$ midguts are bloated and eventually decay. A. Segmentations of µ-CT images of *ctrl*, $ap^{\Delta LSE}$ and ap^{minLSE} one-day-old intestines. The midgut and hindgut are colored in red and green, respectively. The $ap^{\Delta LSE}$ mutant midgut is bloated when compared to *ctrl* and ap^{minLSE} intestines. **B.** Segmentations of µ-CT images of midguts of different genotypes. Removal of the Reinger's knot using byn^{Gal4}>ci^{Rep} rescues the bloated midgut phenotype in $ap^{\Delta LSE}$ flies. **C.** Muscle staining of *ctrl* and $ap^{\Delta LSE}$ mutant intestines with DAPI (blue), Actin (green) and Phalloidin (Phall, red). While no differences are observed in staining patterns, the muscle structure in mutant intestines appears more stretched when compared to *ctrl* intestines. Scale bar: 25 µm. **D.** Overview of larval and adult intestines stained with HRP (red) and Phalloidin (Phall, green). Larval guts of *ctrl* and $ap^{\Delta LSE}$ flies show no differences. However, the midguts of 2-days-old $ap^{\Delta LSE}$ mutants exhibit decay due to a ruptured and discontinuous midgut epithelium, while the intestines of *ctrl* flies are intact. Virgin female flies were used for all experiments. A = anterior. P = posterior. Scale bar: 200 µm.

Figure S11.

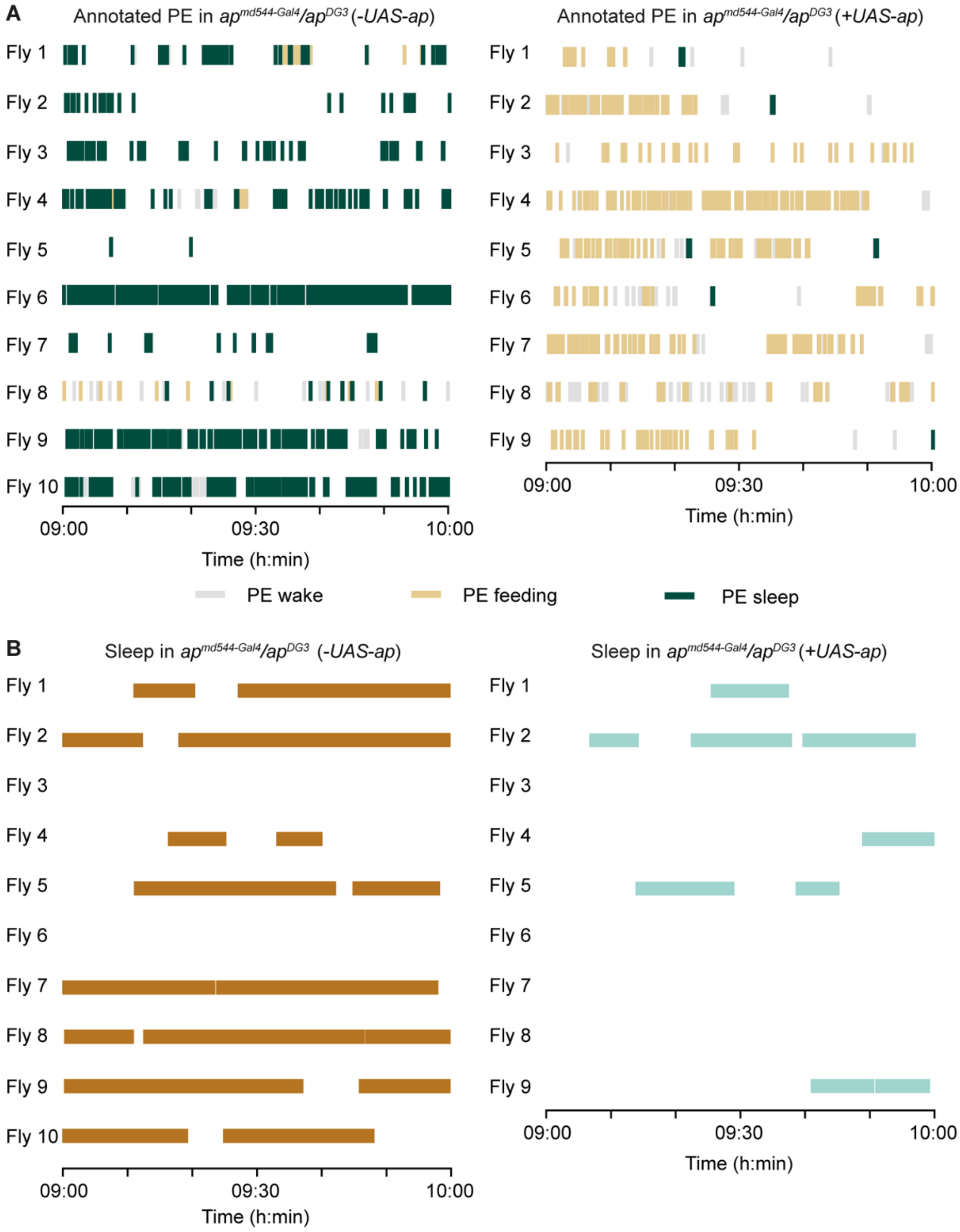

Figure S11. Rasterplots of proboscis extension (PE) events and sleep bouts in *$ap^{md544\text{-}Gal4}$ / ap^{DG3}* flies in the presence or absence of *UAS-ap*. A. Rasterplot of manually annotated PE events for individual flies of the following genotypes: *$ap^{md544\text{-}Gal4}$ / ap^{DG3}* with or without *UAS-ap.* (- *UAS-ap*) flies mainly display PEs during sleep. (+ *UAS-ap*) flies extend their proboscis mostly towards food (the color code for PE wake (while active), PE feeding (towards food) and PE sleep (during sleep) is shown below the plots). **B.** Rasterplot of sleep bouts lasting at least 5 min for *$ap^{md544\text{-}Gal4}$ / ap^{DG3}*flies with or without UAS-*ap*. Virgin female flies were used for all experiments. See Figure 6D-F for quantification.

Figure S12.

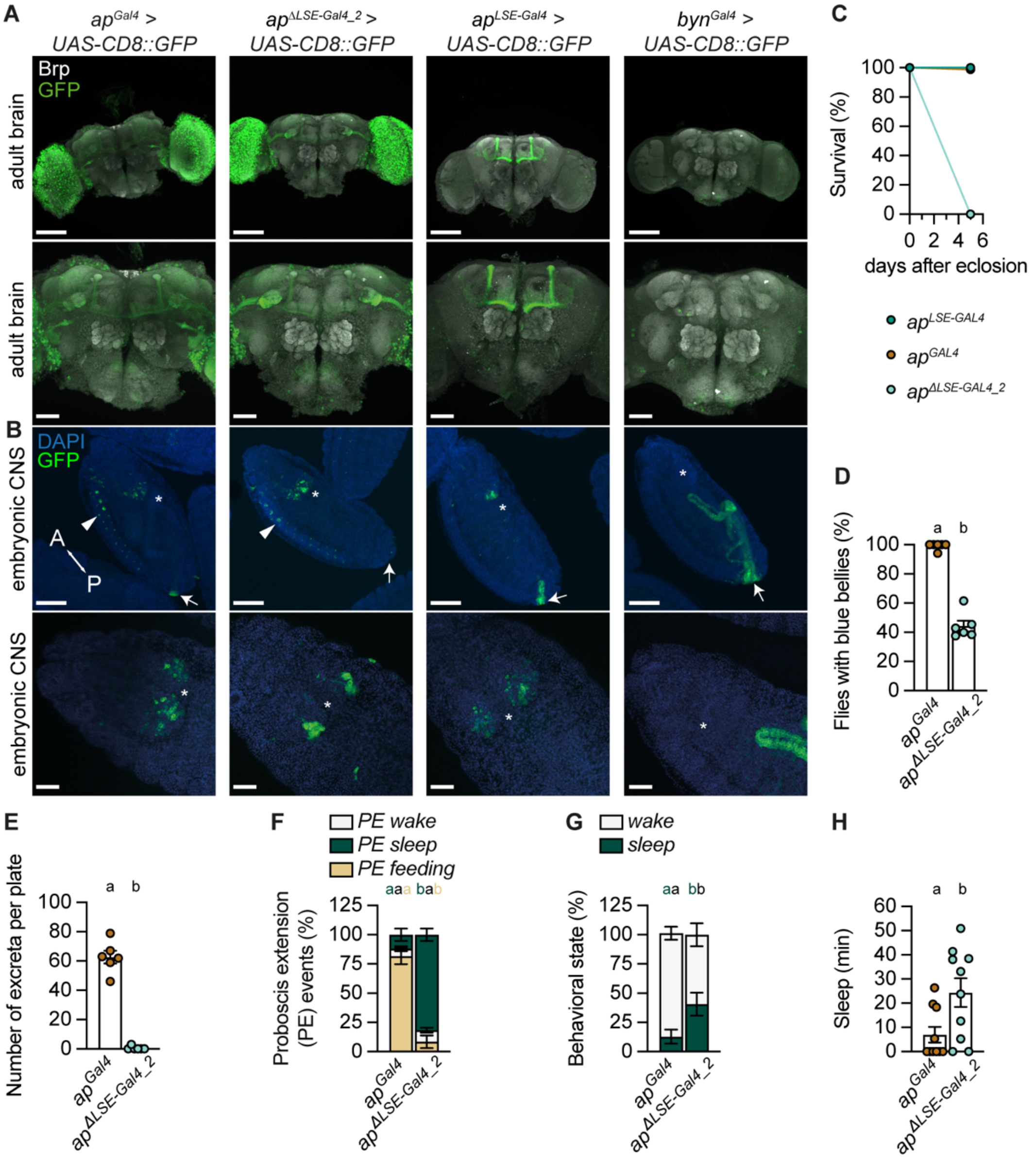

Figure S12. Ap expression in the brain is not sufficient to rescue the survival, feeding initiation, excretion and sleep phenotypes of *ap*$^{\Delta LSE}$ mutants. A. Overview of adult brains expressing CD8::GFP under the control of *ap*^{Gal4}*,* *ap*$^{\Delta LSE\text{-}Gal4\_2}$, *ap*$^{LSE\text{-}Gal4}$ and *byn*^{Gal4}. The Bruchpilot (Brp) marker outlines the adult brain. Top: Whole adult brains. Bottom: Close-up of adult brains. The GFP expression pattern shows no differences in *ap*^{Gal4} and *ap*$^{\Delta LSE\text{-}Gal4\_2}$ adult brains. GFP is mainly expressed in the optic and mushroom body lobes. In *ap*$^{LSE\text{-}Gal4}$ brains, only the mushroom body shows GFP expression. *byn*^{Gal4} flies do not express CD8::GFP in the adult brain. Based on these observations, it is reasonable to assume that the expression pattern of the associated endogenous *ap* and *byn* genes recapitulates the same tissue specificity as the Gal4 driver. These considerations also apply to the data shown in **B**. **B.** Overview of late-stage embryos of *ap*^{Gal4}, *ap*$^{\Delta LSE\text{-}Gal4\_2}$, *ap*$^{LSE\text{-}Gal4}$ and *byn*^{Gal4} flies driving CD8::GFP expression. Top: Whole embryos. Bottom: Close-up of embryonic brain region. In *ap*^{Gal4}, *ap*$^{LSE\text{-}Gal4}$ and *byn*^{Gal4} embryos, CD8::GFP is expressed in the embryonic hindgut (arrows). Importantly, this signal is missing in *ap*$^{\Delta LSE\text{-}Gal4\_2}$ animals. *ap*^{Gal4}, *ap*$^{\Delta LSE\text{-}Gal4\_2}$ and *ap*$^{LSE\text{-}Gal4}$ embryos show additional *CD8::GFP* signal in the embryonic brain (asterisk). *ap*^{Gal4} *and ap*$^{\Delta LSE\text{-}Gal4\_2}$ also initiate the characteristic *ap* VNC pattern (arrow heads; 33).

(**Figure S12 continued**). Differential nervous system expression patterns mediated by ap^{Gal4} *and* $ap^{\Delta LSE\text{-}Gal4\_2}$ on the one hand and $ap^{LSE\text{-}Gal4}$ on the other suggests that *ap* might be regulated by at least two CNS-specific enhancers: one located in the intergenic spacer (*60*), the other an as yet unidentified mushroom body enhancer. byn^{Gal4} embryos exclusively show CD8::GFP expression in the embryonic hindgut. **C-H.** It is important to note that the phenotypes of our ap^{Gal4} drivers (Fig. S1H-K) are not dependent on the presence of a *UAS-ap* transgene. Thus, the phenotypic read-out (survival, ampulla phenotype, behavioral assays; see also Fig. S13) monitors the activity of the associated endogenous *ap* gene. Our observations strongly suggest that these alleles are *bona fide* Gal4 enhancer traps and that the genetic engineering during their generation does not influence the function of the associated *ap* genes. **C.** $ap^{\Delta LSE\text{-}Gal4\_2}$ flies are dying precociously. Thus, nervous system expression detected in $ap^{\Delta LSE\text{-}Gal4\_2}$ flies is unable to mediate adult survival. In contrast, embryonic hindgut expression detected in ap^{Gal4} and $ap^{LSE\text{-}Gal4}$ correlates well with adult survival. Genotype effect: $P < 0.0001$, time x genotype interaction: $P < 0.0001$, RM two-way ANOVA, N = 5-6. **D-H.** Nervous system expression of Ap by $ap^{\Delta LSE\text{-}Gal4\_2}$ does not rescue the feeding (**D**), excretion (**E**), proboscis extension (**F**) and sleep phenotypes (**G,H**). **D:** genotype effect: $P = 0.0022$, Mann Whitney test*,* N = 6; **E:** genotype effect: $P = 0.0022$, Mann Whitney test*,* n = 15, N = 6; **F:** genotype effect: $P = 0.0001$, PERMANOVA*,* n = 10; **G:** genotype effect: $P = 0.0201$, PERMANOVA*,* n = 10; **H:** genotype effect: $P = 0.0289$, Mann Whitney test*,* n = 10. Virgin female flies were used for all experiments except S12B, in which mixed-sex flies were used. Data are means ± s.e.m. n: number of flies. N: number of independent replicates. Groups that do not differ significantly share the same letter, while groups with different letters are statistically significant. For statistical details see Table S1 and for detailed genotype descriptions see Table S2. White: Brp, blue: DAPI, green: GFP (detecting Ap expression). A = anterior, P = posterior. Scale bars = 50 µm.

Figure S13.

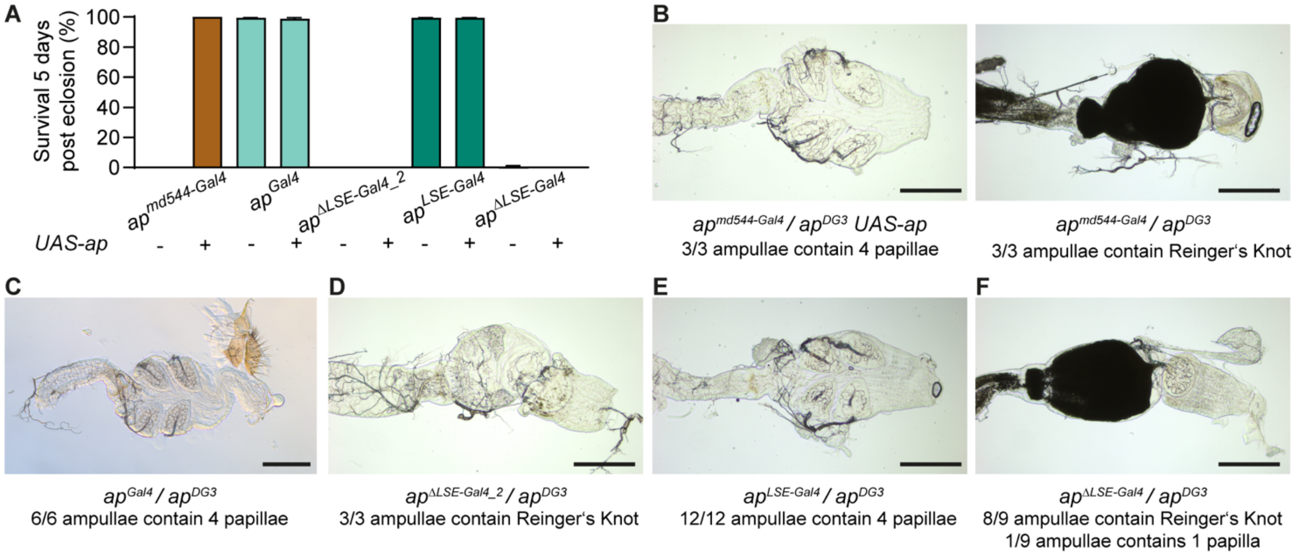

Figure S13. Survival rates and ampulla phenotypes of ap^{Gal4} driver lines in the presence and absence of *UAS-ap*. A. Survival rates of various ap^{Gal4} driver lines are shown in the presence or the absence of *UAS-ap*. In the presence of *UAS-ap*, the precocious death phenotype can be rescued in *ap*$^{md544\text{-}Gal4}$ flies. The function of all other *ap*^{Gal4} alleles is independent of *UAS-ap* because the allele-specific enhancer deletions determine the tissue specific activity of Gal4 as well as of the associated *ap* gene. **B.** The presence of *UAS-ap* leads to normal ampulla formation in *ap*$^{md544\text{-}Gal4}$ flies (left). In the absence of UAS-*ap*, *ap*$^{md544\text{-}Gal4}$ flies form the Reinger's knot (right). **C.** *ap*^{Gal4} ampullae contain four rectal papillae. **D.** The Reinger's knot is formed in *ap*$^{\Delta LSE\text{-}Gal4\_2}$ flies because they lack the LSE. **E.** The presence of the LSE rescues the hindgut phenotype: four papillae are formed in the ampullae of *ap*$^{LSE\text{-}Gal4}$ flies. **F.** As *ap*$^{\Delta LSE\text{-}Gal4\_2}$, *ap*$^{\Delta LSE\text{-}Gal4}$ flies have no functional LSE and therefore form the Reinger's knot. Virgin female flies were used for all experiments except S13A, in which mixed-sex flies were used. Scale bars: 100 µm.

Figure S14.

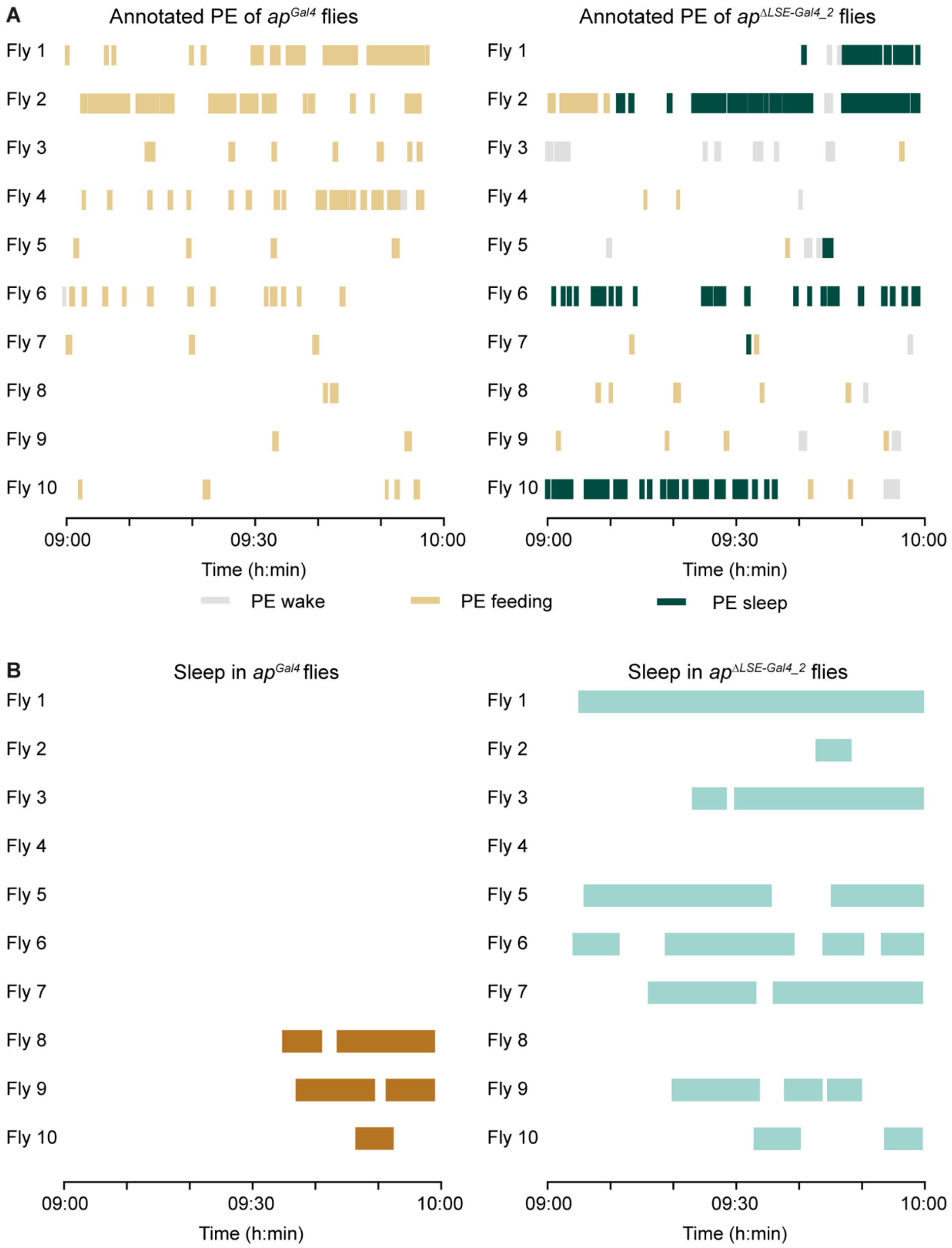

Figure S14. Rasterplots of proboscis extension (PE) events and sleep bouts in ap^{Gal4} and $ap^{\Delta LSE\text{-}Gal4\_2}$ flies. A. Rasterplots of manually annotated PE events for individual ap^{Gal4} (left) and $ap^{\Delta LSE\text{-}Gal4\_2}$ flies (right) are shown. ap^{Gal4} flies tend to extend their proboscis toward food throughout the entire time of recording. $ap^{\Delta LSE\text{-}Gal4\_2}$ flies tend to extend their proboscis toward food or while active at the beginning of the recording and then mainly display PEs during sleep (the color code for PE wake (while active), PE feeding (towards food) and PE sleep (during sleep) is shown below the plots). **B.** Rasterplots of sleep bouts lasting at least 5 min for ap^{Gal4} (left) and or $ap^{\Delta LSE\text{-}Gal4\_2}$ (right) flies. Virgin female flies were used for all experiments. See Figure S12F-H for quantification.

Figure S15.

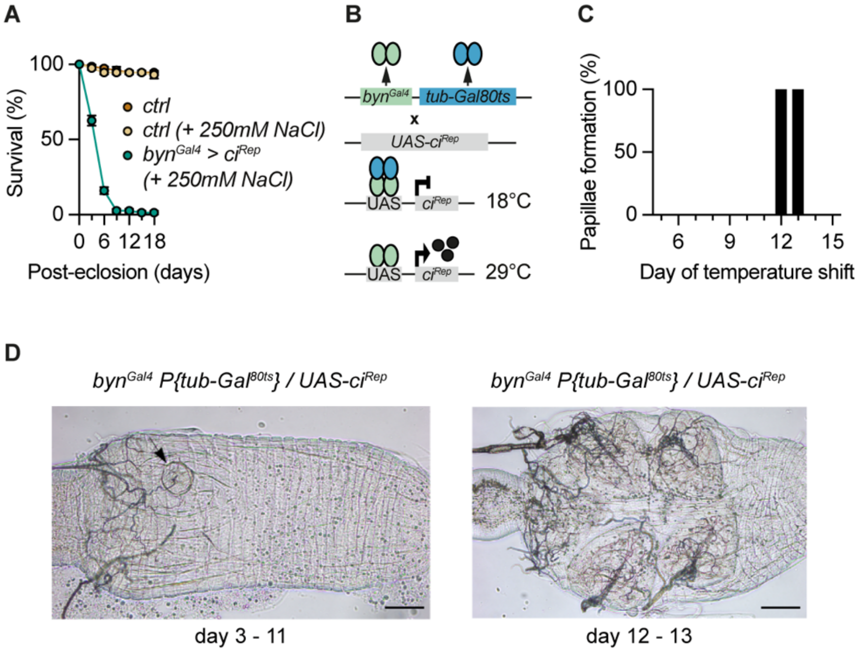

Figure S15. Papillae ablation can be induced by using *byn*^{Gal4}>*ci*^{Rep}. A. Flies lacking papillae (*byn*^{Gal4}>*ci*^{Rep}) cannot survive under high-salt conditions (250 mM NaCl). Genotype effect: $P < 0.0001$, time x genotype interaction: $P < 0.0001$, two-way ANOVA, $N = 3$. Data are means ± s.e.m. N: number of replicates. **B.** The relevant period for *byn*^{Gal4}>*ci*^{Rep} function in papilla ablation was determined through temperature-shift experiments. Schematic overview of the Gal4/Gal80^{ts} system. *byn*^{Gal4} (green) was recombined with *P{tubGal80*^{ts}*}* (blue) and crossed to *UAS-ci*^{Rep} (grey). At 18°C, Gal80^{ts} binds to Gal4 and thus prevents Ci^{Rep} production. At 29°C, Gal80^{ts} is inactive and Gal4 can activate Ci^{Rep} expression. **C.** Animals shifted to 29°C (and hence expressing Ci^{Rep}) before day 12 lack papillae tissue in their ampulla, whereas those shifted after day 11 form papillae (for details, see Table S4). **D.** Ampullae dissected from *byn*^{Gal4} *P{tubGal80*^{ts}*}* > *UAS-ci*^{Rep} females show the absence (left) or presence (right) of papillae tissue when shifted to 29°C before day 12 or after day 11, respectively. In animals shifted on days 3-11, a rudiment of papilla tissue can sometimes be observed. It is still targeted by trachea (arrow, left). Flies shifted after day 12 usually contain four papillae within their rectal ampulla, each papilla targeted by an organized trachea network. Virgin female flies were used for all experiments except S15A, in which mixed-sex flies were used. For statistical details see Table S1 and for detailed genotype descriptions see Table S2.

Figure S16.

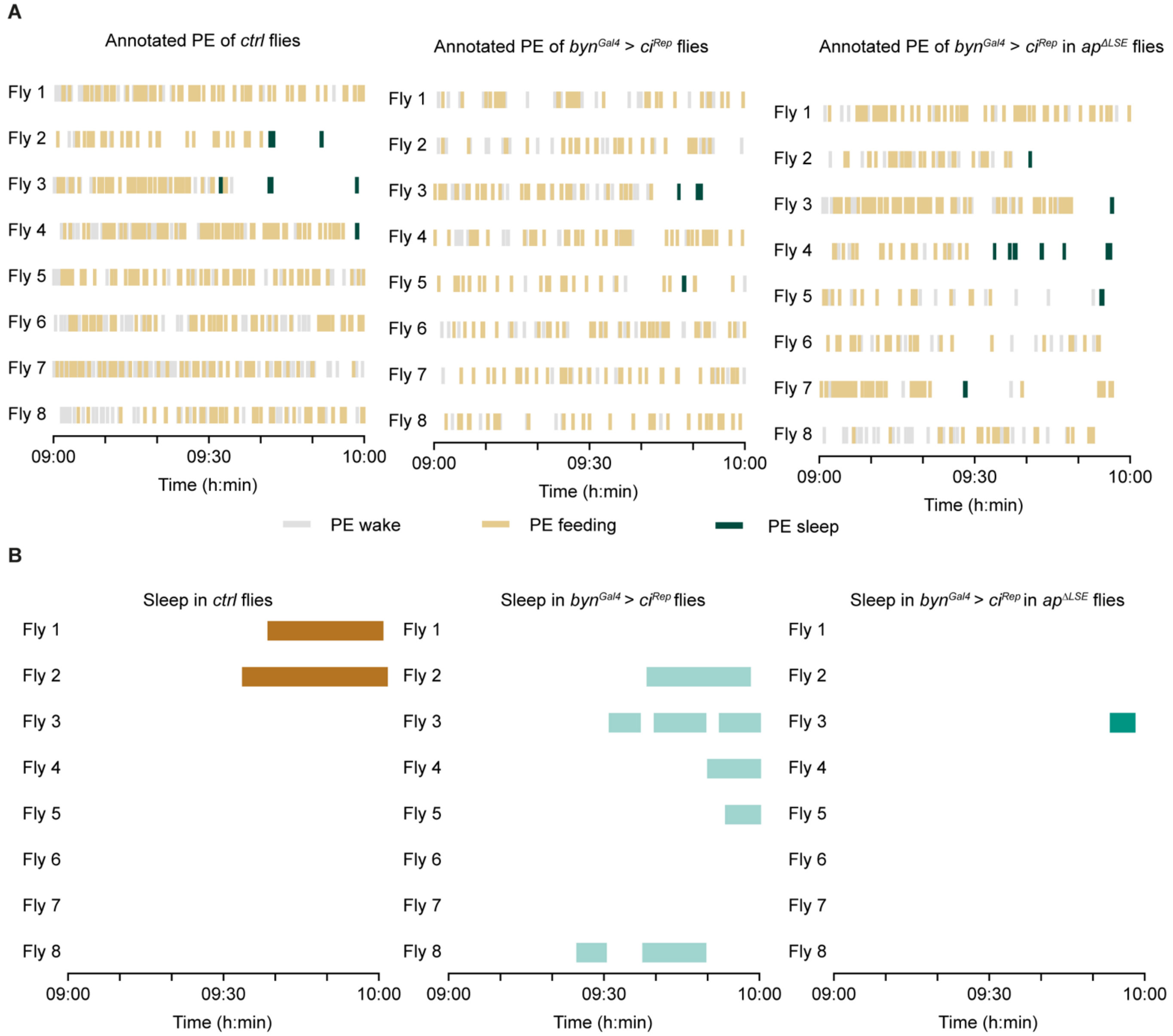

Figure S16. Rasterplots of proboscis extension (PE) events and sleep bouts in flies without papillae tissue. A. Rasterplots of manually annotated PE events for individual *ctrl* (left), *byn*^{Gal4} >*ci*^{Rep} (middle) and *byn*^{Gal4} >*ci*^{Rep} in *ap*$^{\Delta LSE}$ (right) flies. All flies perform almost all PE events toward food or while active during one hour of recording. The color code for PE wake (while active), PE feeding (towards food) and PE sleep (during sleep) is shown below the plots. **B.** Rasterplots of sleep bouts lasting at least 5 min for individual *ctrl* (left), *byn*^{Gal4} >*ci*^{Rep} (middle) and *byn*^{Gal4} >*ci*^{Rep} in *ap*$^{\Delta LSE}$ (right) flies. Virgin female flies were used for all experiments. See Figure 7F-H for quantification.

Figure S17.

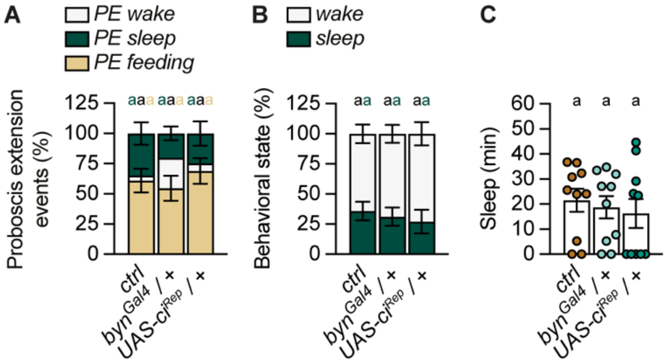

Figure S17. Quantification of proboscis extension (PE) events and sleep in *byn^{Gal4}* / + and *UAS-ci^{Rep}* flies. A. Annotated proboscis extension (PE) ratios during wake, sleep or towards food over a 1 h recording show that all three genotypes behave the same and perform most of their PEs toward food. Genotype effect: $P = 0.29$, PERMANOVA, $n = 10$. **B.** All three genotypes spend more time awake than asleep. Genotype effect: $P = 0.80$, PERMANOVA, $n = 10$. **C.** Total sleep quantification shows no differences among all three genotypes. Genotype effect: $P = 0.06604$, Kruskal Wallis ANOVA, $n = 10$. Virgin female flies were used for all experiments. Data are means ± s.e.m. *n*: number of flies. Groups that do not differ significantly share the same letter, while groups with different letters are statistically significant. For statistical details see Table S1 and for detailed genotype descriptions see Table S2.

Figure S18.

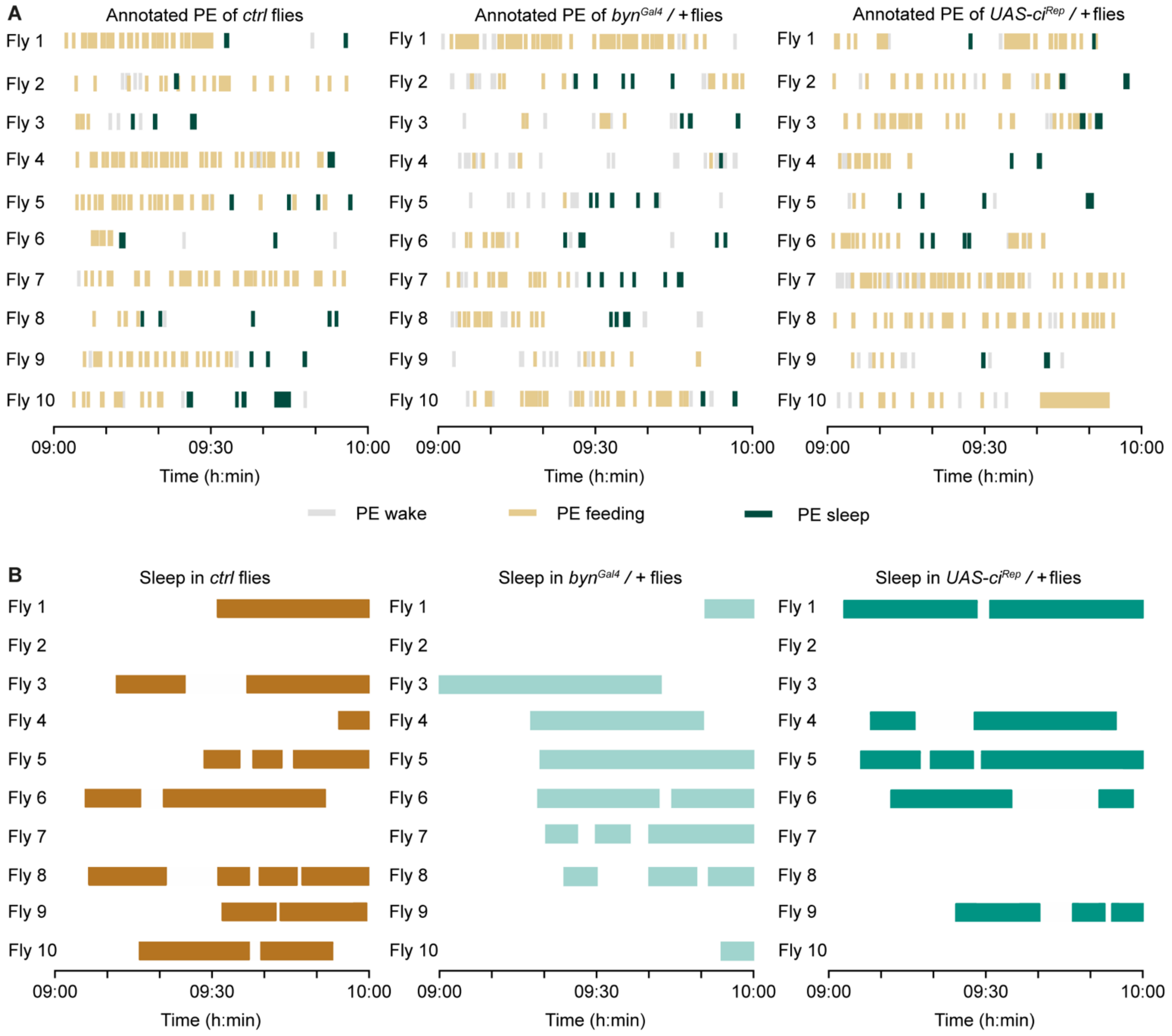

Figure S18. Rasterplots of proboscis extension (PE) events and sleep bouts in *byn*^{Gal4} / + and *UAS-ci*^{Rep} flies. A. Rasterplots of manually annotated PE events (the color code for PE wake (while active), PE feeding (towards food) and PE sleep (during sleep) is shown below the plots) for individual *ctrl* (left), *byn*^{Gal4} / + (middle) and *UAS-ci*^{Rep} / + (right) flies. Constipated flies tend to extend their proboscis toward food or while active at the beginning of the recording and then mainly display proboscis extension during sleep. **B.** Rasterplot of sleep bouts lasting at least 5 min for individual *ctrl* (left), *byn*^{Gal4} / + (middle) and *UAS-ci*^{Rep} / + (right) flies. Virgin female flies were used for all experiments. See Figure S17 for quantification.

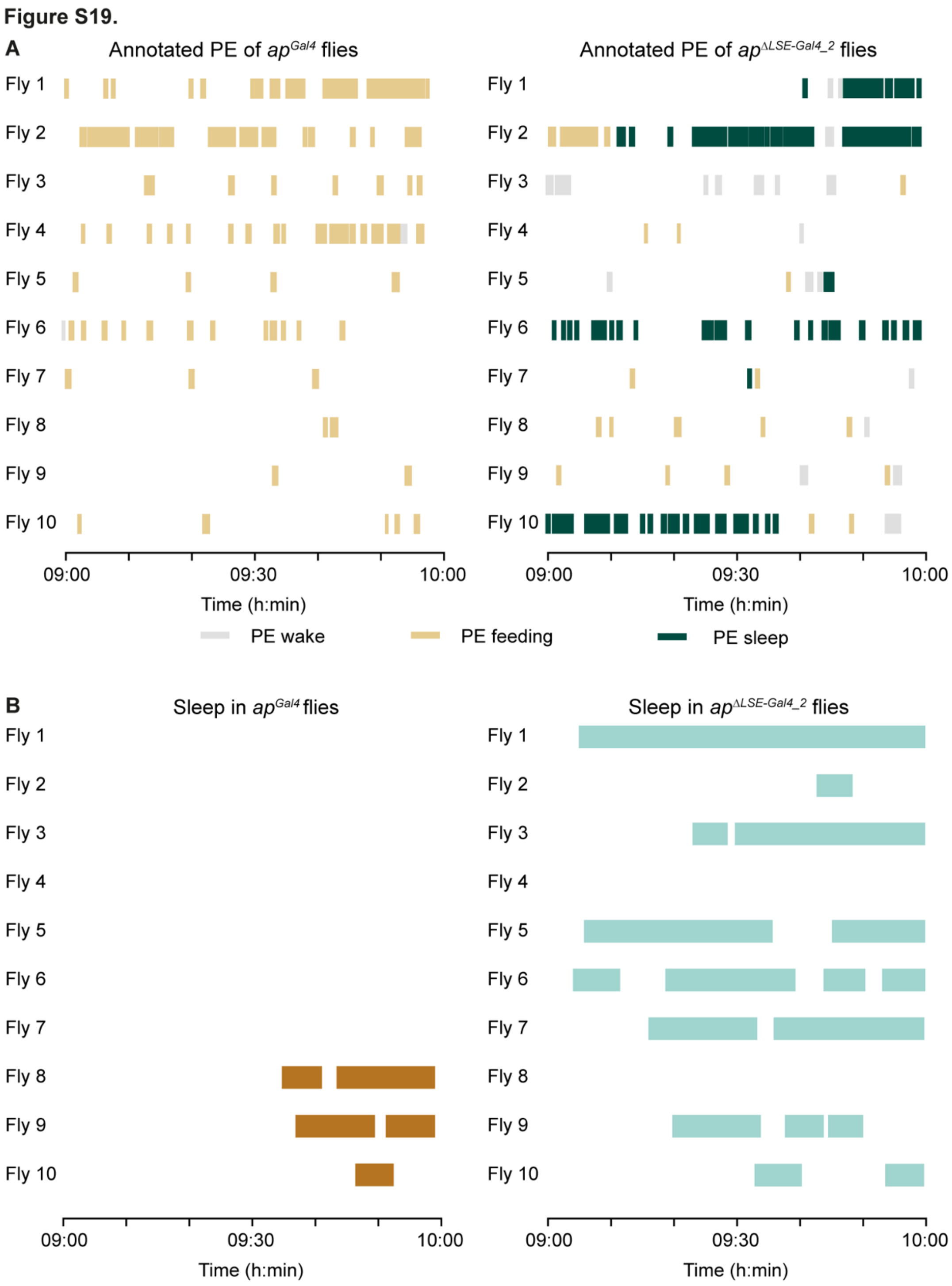

Figure S19. Rasterplots of proboscis extension (PE) events and sleep bouts in *ctrl* flies with a sealed anus. A. Rasterplots of manually annotated PE events for individual *ctrl* (left) and *ctrl* flies with a sealed anus (right). The color code for PE wake (while active), PE feeding (towards food) and PE sleep (during sleep) is shown below the plots. Constipated flies tend to extend their proboscis toward food or while active at the beginning of the recording and then mainly display proboscis extension during sleep. Color code for PE wake (while active), PE feeding (towards food) and PE sleep (during sleep) is indicated below the plots. **B.** Rasterplot of sleep bouts lasting at least 5 min for individual *ctrl* (left) or *ctrl* with a sealed anus (right) flies. Virgin female flies were used for all experiments. See Figure 8E-G for quantification.

Table S1. Statistical analyses.

Figure	Statistical test	Pairwise comparison	Test statistic	*P*
**1B**	Two-way ANOVA			
	Time		$F_{6,28} = 213.3$	<0.0001
	Gender		$F_{1,28} = 5.363$	0.0281
	Time x Gender		$F_{6,28} = 2.146$	0.0792
**1C**	Two-way RM ANOVA			
	Time		$F_{1.307,7.841} = 275.2$	<0.0001
	Excreta type		$F_{1,6} = 78.05$	0.0001
	Time x Excreta type		$F_{1.307,7.841} = 11.25$	0.0078
**1D**	Mixed-effects model			
	Time		$F_{2.247,13.07} = 45.43$	<0.0001
	Gender		$F_{1,6} = 22.42$	0.0032
	Time x Gender		$F_{2.247,13.07} = 4.350$	0.0323
**1E**	Two-way ANOVA			
	Time		$F_{3,24} = 1958$	<0.0001
	Food		$F_{1,24} = 4270$	<0.0001
	Time x Food		$F_{3,24} = 1083$	<0.0001
**1F**	Two-way ANOVA			
	Time		$F_{3,24} = 2566$	<0.0001
	Food		$F_{1,24} = 5161$	<0.0001
	Time x Food		$F_{3,24} = 1395$	<0.0001
**1G**	Two-way RM ANOVA			
	Time		$F_{1.000,4.000} = 9663$	<0.0001
	Age		$F_{1.000,4.000} = 2489$	<0.0001
	Time x Age		$F_{3,16} = 2489$	<0.0001
**1H**	Two-way RM ANOVA			
	Time		$F_{1.000,4.000} = 7902$	<0.0001
	Age		$F_{1,4} = 2071$	<0.0001
	Time x Age		$F_{1.000,4.000} = 2071$	<0.0001
**2B**	Two-way ANOVA			
	Time		$F_{6,77} = 153.5$	<0.0001
	Genotype		$F_{2,77} = 3853$	<0.0001
	Time x Genotype		$F_{12,77} = 108.7$	<0.0001
**2C**	Kruskal Wallis ANOVA		U = 11.82	0.0003
	Dunn's post-hoc test	*ctrl vs.* $ap^{\Delta LSE}$	Z = 2.767	0.0170
	Dunn's post-hoc test	*ctrl vs.* ap^{minLSE}	Z = 0.3835	>0.9999
	Dunn's post-hoc test	$ap^{\Delta LSE}$ *vs.* ap^{minLSE}	Z = 3.150	0.0049
**2D**	Kruskal Wallis ANOVA		U = 11.68	0.0029
	Dunn's post-hoc test	*ctrl vs.* $ap^{\Delta LSE}$	Z = 3.007	0.0079
	Dunn's post-hoc test	*ctrl vs.* ap^{minLSE}	Z = 0.02071	>0.9999
	Dunn's post-hoc test	$ap^{\Delta LSE}$ *vs.* ap^{minLSE}	Z = 2.869	0.0124
**2E**	Kruskal Wallis ANOVA		U = 47.08	<0.0001
	Dunn's post-hoc test	*ctrl vs.* $ap^{\Delta LSE}$	Z = 6.834	<0.0001
	Dunn's post-hoc test	*ctrl vs.* ap^{minLSE}	Z = 3.150	0.0049
	Dunn's post-hoc test	$ap^{\Delta LSE}$ *vs.* ap^{minLSE}	Z = 3.545	0.0012
**3A**	PERMANOVA		$F_{2,38} = 37.06$	<0.0001
	Tuckey's post-hoc test			
	PE wake	*ctrl vs.* $ap^{\Delta LSE}$		0.0284
		ctrl vs. ap^{minLSE}		0.2846
		$ap^{\Delta LSE}$ *vs.* ap^{minLSE}		0.4814
	PE sleep	*ctrl vs.* $ap^{\Delta LSE}$		<0.0001
		ctrl vs. ap^{minLSE}		0.0551
		$ap^{\Delta LSE}$ *vs.* ap^{minLSE}		<0.0001
	PE feeding	*ctrl vs.* $ap^{\Delta LSE}$		<0.0001
		ctrl vs. ap^{minLSE}		0.2575
		$ap^{\Delta LSE}$ *vs.* ap^{minLSE}		<0.0001
**3B**	PERMANOVA		$F_{2,38} = 13.93$	<0.0001
	Tuckey's post-hoc test			
	PE wake	*ctrl vs.* $ap\Delta^{LSE}$		0.0001
		ctrl vs. ap^{minLSE}		0.7625
		$ap^{\Delta LSE}$ *vs.* ap^{minLSE}		0.0004
	PE sleep	*ctrl vs.* $ap^{\Delta LSE}$		0.0001
		ctrl vs. ap^{minLSE}		0.7625
		$ap^{\Delta LSE}$ *vs.* ap^{minLSE}		0.0004
**3C**	Kruskal Wallis ANOVA		U = 16.49	0.0003
	Dunn's post-hoc test	*ctrl vs.* $ap^{\Delta LSE}$	Z = 3.805	0.0004
	Dunn's post-hoc test	*ctrl vs.* ap^{minLSE}	Z = 0.7602	>0.9999
	Dunn's post-hoc test	$ap^{\Delta LSE}$ *vs.* ap^{minLSE}	Z = 3.103	0.0058
**3D**	PERMANOVA		$F_{1,18} = 2.39$	0.088
**3E**	PERMANOVA		$F_{1,18} = 0.02$	0.0867
**6B**	Two-way ANOVA			
	Time		$F_{6,42} = 185.5$	<0.0001
	Pulse		$F_{1,42} = 4826$	<0.0001
	Time x Pulse		$F_{6,42} = 145.0$	<0.0001
**6C**	Mann-Whitney test		U = 0	0.0286
**6D**	PERMANOVA		$F_{1,17} = 100.18$	<0.0001
	Tuckey's post-hoc test			
	PE wake	*pulse vs. no pulse*		0.0996
	PE sleep	*pulse vs. no pulse*		0.0006
	PE feeding	*pulse vs. no pulse*		0.0012
**6E**	PERMANOVA		$F_{1,17} = 10.27$	0.006
	Tuckey's post-hoc test			
	PE wake	*pulse vs. no pulse*		0.01036
	PE sleep	*pulse vs. no pulse*		0.01036

**6F**	Mann-Whitney test		U = 16	0.0144
**7D**	Two-way ANOVA<br>Time<br>Genotype<br>Time x Genotype		 $F_{6,56}$ = 184.2<br>$F_{3,56}$ = 4428<br>$F_{18,56}$ = 124.4	 <0.0001<br><0.0001<br><0.0001
**7E**	Kruskal Wallis ANOVA<br>Dunn's post-hoc test<br>Dunn's post-hoc test<br>Dunn's post-hoc test	 *ctrl vs. byn^{Gal4} in ap$^{\Delta LSE}$*<br>*ctrl* vs. *byn^{Gal4} > ci^{Rep} in ap$^{\Delta LSE}$*<br>*byn^{Gal4} in ap$^{\Delta LSE}$ vs. byn^{Gal4} > ci^{Rep} in ap$^{\Delta LSE}$*	U = 11.23<br>Z = 3.023<br>Z = 0.1328<br>Z = 2.799	0.0012<br>0.0075<br>>0.9999<br>0.0154
**7F**	PERMANOVA		$F_{2,21}$ = 0.80	0.543
**7G**	PERMANOVA		$F_{2,21}$ = 2.24	0.132
**7H**	Kruskal Wallis ANOVA		U = 4.282	0.1175
**8B**	Two-way ANOVA<br>Time<br>Phenotype<br>Time x Phenotype		 $F_{6,63}$ = 273.1<br>$F_{2,63}$ = 3340<br>$F_{12,63}$ = 195.6	 <0.0001<br><0.0001<br><0.0001
**8D**	Mann-Whitney test		U = 0	0.0286
**8E**	PERMANOVA<br>Tuckey's post-hoc test<br>*PE wake*<br>*PE sleep*<br>*PE feeding*	 <br>*ctrl vs. anus-glued*<br>*ctrl vs. anus-glued*<br>*ctrl vs. anus-glued*	$F_{1,14}$ = 56.37	0.0002<br><br>0.0996<br>0.0006<br>0.0012
**8F**	PERMANOVA<br>Tuckey's post-hoc test<br>*PE wake*<br>*PE sleep*	 <br>*pulse vs. no pulse*<br>*pulse vs. no pulse*	$F_{1,14}$ = 8.02	0.0104<br><br>0.0266<br>0.0266
**8G**	Mann-Whitney test		U = 10	0.0145
**S2A**	Kruskal Wallis ANOVA<br>Dunn's post-hoc test<br>Dunn's post-hoc test<br>Dunn's post-hoc test	 *ctrl vs. ap$^{\Delta LSE}$*<br>*ctrl vs. anus-glued*<br>*ap$^{\Delta LSE}$ vs. anus-glued*	U = 12.96<br>Z = 2.952<br>Z = 3.245<br>Z = 0.3888	0.0015<br>0.0095<br>0.0035<br>>0.9999
**S6A**	PERMANOVA<br>Tuckey's post-hoc test<br>*PE wake*<br>*PE sleep*<br>*PE feeding*	 <br>*ctrl vs. ap$^{\Delta LSE}$*<br>*ctrl vs. ap$^{\Delta LSE}$*<br>*ctrl vs. ap$^{\Delta LSE}$*	$F_{1,25}$ = 64.6	<0.0001<br><br>0.0085<br><0.0001<br><0.0001
**S6B**	PERMANOVA<br>Tuckey's post-hoc test<br>*PE wake*<br>*PE sleep*<br>*PE feeding*	 <br>*ctrl vs. ap$^{\Delta LSE}$*<br>*ctrl vs. ap$^{\Delta LSE}$*<br>*ctrl vs. ap$^{\Delta LSE}$*	$F_{1,18}$ = 4.013	0.03<br><br>0.5777<br>0.5259<br>0.5777
**S7**	Kruskal Wallis ANOVA<br>Dunn's post-hoc test<br>Dunn's post-hoc test<br>Dunn's post-hoc test<br>Dunn's post-hoc test<br>Dunn's post-hoc test<br>Dunn's post-hoc test	 *ctrl (5d) vs. ctrl (1d)*<br>*ctrl (5d) vs. ap$^{\Delta LSE}$ (1d)*<br>*ctrl (5d) vs. ctrl (1d starved)*<br>*ctrl (1d) vs. ap$^{\Delta LSE}$ (1d)*<br>*ctrl (1d) vs. ctrl (1d starved)*<br>*ap$^{\Delta LSE}$ (1d) vs. ctrl (1d starved)*	U = 59.39<br>Z = 2.632<br>Z = 5.276<br>Z = 1.986<br>Z = 2.681<br>Z = 4.619<br>Z = 7.234	<0.0001<br>0.0509<br><0.0001<br>0.2819<br>0.0440<br><0.0001<br><0.0001
**S8A**	Mann-Whitney test		U = 11	0.0021
**S8B**	Mann-Whitney test		U = 0	0.0004
**S12C**	Two-way RM ANOVA<br>Time<br>Genotype<br>Time x Genotype		 $F_{1,13}$ = 23858<br>$F_{2,13}$ = 24504<br>$F_{2,13}$ = 24504	 <0.0001<br><0.0001<br><0.0001
**S12D**	Mann-Whitney test		U = 0	0.0022
**S12E**	Mann-Whitney test		U = 0	0.0022
**S12F**	PERMANOVA<br>Tuckey's post-hoc test<br>*PE wake*<br>*PE sleep*<br>*PE feeding*	 <br>*ap^{Gal4} > UAS-ap vs. ap$^{\Delta LSE\text{-}Gal4_2}$ > UAS-ap*<br>*ap^{Gal4} > UAS-ap vs. ap$^{\Delta LSE\text{-}Gal4_2}$ > UAS-ap*<br>*ap^{Gal4} > UAS-ap vs. ap$^{\Delta LSE\text{-}Gal4_2}$ > UAS-ap*	$F_{1,18}$ = 71.20	0.0001<br><br>0.7846<br>0.0004<br>0.0002
**S12G**	PERMANOVA<br>Tuckey's post-hoc test<br>*PE wake*<br>*PE sleep*	 <br>*ap^{Gal4} > UAS-ap vs. ap$^{\Delta LSE\text{-}Gal4_2}$ > UAS-ap*<br>*ap^{Gal4} > UAS-ap vs. ap$^{\Delta LSE\text{-}Gal4_2}$ > UAS-ap*	$F_{1,18}$ = 6.62	0.0201<br><br>0.0383<br>0.0383
**S12H**	Mann-Whitney test		U = 22	0.0289
**S15A**	Two-way ANOVA<br>Time<br>Genotype<br>Time x Genotype		 $F_{6,42}$ = 243.3<br>$F_{2,42}$ = 4326<br>$F_{12,42}$ = 176.1	 <0.0001<br><0.0001<br><0.0001
**S17A**	PERMANOVA		$F_{2,27}$ = 1.19	0.29
**S17B**	PERMANOVA		$F_{2,27}$ = 0.2	0.80
**S17C**	Kruskal Wallis ANOVA		U = 0.8299	0.06604

Table S2. Detailed description of genotypes. *N* or *n*: number of independent replicates. For some experiments, each replicate represents the average of multiple flies; in these cases, the number of flies per replicate is indicated in parentheses.

Figure	Name in Figure	genotype	***N* or *n***
**1B**	*ctrl females*	*y w*	3 (25-30 flies each)
	ctrl males	*y w*	3 (25-30 flies each)
**1C**	*meconium*	*y w*	4 (13-19 flies each)
	blue food	*y w*	4 (13-19 flies each)
**1D**	*ctrl females*	*y w*	4 (10-19 flies each)
	ctrl males	*y w*	4 (10-19 flies each)
**1E**	*ctrl females (- food)*	*y w*	4 (24-25 flies each)
	ctrl females (+ food)	*y w*	4 (24-25 flies each)
**1F**	*ctrl males (- food)*	*y w*	4 (25 flies each)
	ctrl males (+ food)	*y w*	4 (25 flies each)
**1G**	*ctrl females (5 days old)*	*y w*	3 (20-25 flies each)
	ctrl females (juvenile)	*y w*	3 (20-25 flies each)
**1H**	*ctrl males (5 days old)*	*y w*	3 (20-25 flies each)
	ctrl males (juvenile)	*y w*	3 (20-25 flies each)
**2B**	*ctrl*	*y w*	4 (25 flies each)
	$ap^{\Delta LSE}$	y w ; ap^{MS2} / ap^{DG3}	5 (19-24 flies each)
	ap^{minLSE}	y w ; $ap^{MS2+minLSE}$ / ap^{DG3}	5 (15-20 flies each)
**2C**	*ctrl*	*y w*	6 (12-14 flies each)
	$ap^{\Delta LSE}$	y w ; ap^{MS2} / ap^{DG3}	6 (10-13 flies each)
	ap^{minLSE}	y w ; $ap^{MS2+minLSE}$ / ap^{DG3}	6 (10-13 flies each)
**2D**	*ctrl*	*y w*	14 (3 flies each)
	$ap^{\Delta LSE}$	y w ; ap^{MS2} / ap^{DG3}	14 (3 flies each)
	ap^{minLSE}	y w ; $ap^{MS2+minLSE}$ / ap^{DG3}	12 (3 flies each)
**2E**	*ctrl*	*y w*	30
	$ap^{\Delta LSE}$	y w ; ap^{MS2} / ap^{DG3}	39
	ap^{minLSE}	y w ; $ap^{MS2+minLSE}$ / ap^{DG3}	31
**2F**	ap^{minLSE}	y w ; $ap^{MS2+minLSE}$ / ap^{DG3}	
	$ap^{\Delta LSE}$	y w ; ap^{MS2} / ap^{DG3}	
**3A-C**	*ctrl*	*y w*	13
	$ap^{\Delta LSE}$	y w ; ap^{MS2} / ap^{DG3}	14
	ap^{minLSE}	y w ; $ap^{MS2+minLSE}$ / ap^{DG3}	14
**3D-E**	fed	*y w*	8
	starved	*y w*	12
**4B**	byn^{Gal4} > $mCherry^{NLS}$	y w ; byn^{Gal4} UAS-$mCherry^{NLS}$ / +	
**4C**	ap^{Gal4} > G-TRACE	y w ; $ap^{c1.4b\text{-}Gal4}$ / ap^{DG3} ; G-TRACE / +	
	$ap^{\Delta LSE\text{-}Gal4}$ > G-TRACE	y w ; $ap^{DG1\text{-}Gal4}$ / ap^{DG3} ; G-TRACE / +	
	$ap^{LSE\text{-}Gal4}$ > G-TRACE	y w ; $ap^{R1+LSE\text{-}Gal4}$ / ap^{DG3} ; G-TRACE / +	
**4D**	*ctrl*	*y w*	
	$ap^{\Delta LSE}$	y w ; ap^{MS2} / ap^{DG3}	
	ap^{minLSE}	y w ; $ap^{MS2+minLSE}$ / ap^{DG3}	
**5B**	*ctrl*	*y w*	
	$ap^{\Delta LSE}$	y w ; ap^{MS2} / ap^{DG3}	
**5C**	Dl^{Gal4} >$mCherry^{NLS}$ in *ctrl*	y w ; Dl^{Gal4} UAS-$mCherry^{NLS}$ / +	
	Dl^{Gal4} >$mCherry^{NLS}$ in $ap^{\Delta LSE}$	y w ; ap^{MS2} / ap^{DG3} ; Dl^{Gal4} UAS-$mCherry^{NLS}$ / +	
**5E**		y w ; ap^{md544} tub-$Gal80^{ts}$ / ap^{DG3} UAS-ap	10-20 (Table S3)
**5G**	*ctrl*	*y w*	
	$ap^{\Delta LSE}$	y w ; ap^{MS2} / ap^{DG3}	
**6A**	$ap^{md544\text{-}Gal4}$/ ap^{DG3} (-UAS-ap)	y w ; ap^{md544} tub-$Gal80^{ts}$ / ap^{DG3} P{UAS-CD8-GFP}	
	$ap^{md544\text{-}Gal4}$/ ap^{DG3} (+UAS-ap)	y w ; ap^{md544} tub-$Gal80^{ts}$ / ap^{DG3} UAS-ap P{UAS-CD8-GFP}	
**6B**	$ap^{md544\text{-}Gal4}$/ ap^{DG3} (-UAS-ap)	y w ; ap^{md544} tub-$Gal80^{ts}$ / ap^{DG3} P{UAS-CD8-GFP}	4 (15-22 flies each)
	$ap^{md544\text{-}Gal4}$/ ap^{DG3} (+UAS-ap)	y w ; ap^{md544} tub-$Gal80^{ts}$ / ap^{DG3} UAS-ap P{UAS-CD8-GFP}	4 (20-24 flies each)
**6C**	*- UAS-ap*	y w ; ap^{md544} tub-$Gal80^{ts}$ / ap^{DG3} P{UAS-CD8-GFP}	4 (17-23 flies each)
	+ UAS-ap	y w ; ap^{md544} tub-$Gal80^{ts}$ / ap^{DG3} UAS-ap P{UAS-CD8-GFP}	4 (15-22 flies each)
**6D-F**	*- UAS-ap*	y w ; ap^{md544} tub-$Gal80^{ts}$ / ap^{DG3} P{UAS-CD8-GFP}	10
	+ UAS-ap	y w ; ap^{md544} tub-$Gal80^{ts}$ / ap^{DG3} UAS-ap P{UAS-CD8-GFP}	9
**7A**	*ctrl*	*y w*	
	ci^{Rep}	y w ; UAS-ci^{Rep} / +	
	byn^{Gal4}	y w ; byn^{Gal4} / +	
	byn^{Gal4} > ci^{Rep}	y w ; byn^{Gal4} UAS-ci^{Rep} / +	
**7B**	byn^{Gal4}	y w ; byn^{Gal4} / +	
	byn^{Gal4} in $ap^{\Delta LSE}$	y w ; ap^{MS2} / ap^{DG3} ; byn^{Gal4} / +	
	byn^{Gal4} > ci^{Rep}	y w ; byn^{Gal4} UAS-ci^{Rep} / +	
	byn^{Gal4} > ci^{Rep} in $ap^{\Delta LSE}$	y w ; ap^{MS2} / ap^{DG3} ; byn^{Gal4} UAS-ci^{Rep} / +	
**7D**	*ctrl*	*y w*	3 (25 flies each)
	byn^{Gal4} in $ap^{\Delta LSE}$	y w ; ap^{MS2} / ap^{DG3} ; byn^{Gal4} / +	3 (22-26 flies each)
	byn^{Gal4} > ci^{Rep}	y w ; byn^{Gal4} UAS-ci^{Rep} / +	3 (23-27 flies each)
	byn^{Gal4} > ci^{Rep} in $ap^{\Delta LSE}$	y w ; ap^{MS2} / ap^{DG3} ; byn^{Gal4} UAS-ci^{Rep} / +	3 (22-25 flies each)
**7E**	*ctrl*	*y w*	8 (3 flies each)
	byn^{Gal4} in $ap^{\Delta LSE}$	y w ; ap^{MS2} / ap^{DG3} ; byn^{Gal4} / +	7 (3 flies each)
	byn^{Gal4} > ci^{Rep}	y w ; byn^{Gal4} UAS-ci^{Rep} / +	7 (3 flies each)
**7F-H**	*ctrl*	*y w*	8
	byn^{Gal4} > ci^{Rep}	y w ; byn^{Gal4} UAS-ci^{Rep} / +	8
	byn^{Gal4} > ci^{Rep} in $ap^{\Delta LSE}$	y w ; ap^{MS2} / ap^{DG3} ; byn^{Gal4} UAS-ci^{Rep} / +	8
**8B**	*ctrl*	*y w*	4 (25 flies each)
	sham-glued	*y w*	3 (10-15 flies each)
	anus-glued	*y w*	3 (10-17 flies each)
**8C**	*ctrl*	*y w*	
	anus-glued	*y w*	
**8D**	*ctrl*	*y w*	4 (15 flies each)
	anus-glued	*y w*	4 (15 flies each)
**8E-G**	*ctrl*	*y w*	8
	anus-glued	*y w*	8
**S2A**	*ctrl*	*y w*	33
		y w ; ap^{MS2} / ap^{DG3}	34

	$ap^{\Delta LSE}$<br>*anus-glued*	*y w*	30
**S2B**	*ctrl*<br>$ap^{\Delta LSE}$<br>*anus-glued*	*y w*<br>*y w* ; ap^{MS2} / ap^{DG3}<br>*y w*	33<br>34<br>30
**S2C-E**	*ctrl*<br>$ap^{\Delta LSE}$<br>*anus-glued*	*y w*<br>*y w* ; ap^{MS2} / ap^{DG3}<br>*y w*	
**S3A-B**	*ctrl*<br>$ap^{\Delta LSE}$<br>ap^{minLSE}	*y w*<br>*y w* ; ap^{MS2} / ap^{DG3}<br>*y w* ; $ap^{MS2+minLSE}$ / ap^{DG3}	13<br>14<br>14
**S4**	*ctrl*<br>$ap^{\Delta LSE}$	*y w*<br>*y w* ; ap^{MS2} / ap^{DG3}	13<br>14
**S5A-B**	*ctrl* fed<br>*ctrl* starved	*y w*<br>*y w*	8<br>12
**S6A**	*ctrl*<br>$ap^{\Delta LSE}$	*y w*<br>*y w* ; ap^{MS2} / ap^{DG3}	13<br>14
**S6B**	*ctrl*<br>$ap^{\Delta LSE}$	*y w*<br>*y w* ; ap^{MS2} / ap^{DG3}	10<br>10
**S7**	*ctrl* (1 day) *ad libitum*<br>$ap^{\Delta LSE}$ (1 day) *ad libitum*<br>*ctrl* (5 days) *ad libitum*<br>*ctrl* (1 day) starved	*y w*<br>*y w* ; ap^{MS2} / ap^{DG3}<br>*y w*<br>*y w*	18<br>17<br>18<br>18
**S8A**	fed<br>starved	*y w*<br>*y w*	10<br>10
**S8B**	*ctrl*<br>$ap^{\Delta LSE}$	*y w*<br>*y w* ; ap^{MS2} / ap^{DG3}	6<br>9
**S9A-B**	*ctrl*<br>$ap^{\Delta LSE}$	*y w*<br>*y w* ; ap^{MS2} / ap^{DG3}	
**S10A**	*ctrl*<br>$ap^{\Delta LSE}$<br>ap^{minLSE}	*y w*<br>*y w* ; ap^{MS2} / ap^{DG3}<br>*y w* ; $ap^{MS2+minLSE}$ / ap^{DG3}	
**S10B**	byn^{Gal4} in $ap^{\Delta LSE}$<br>$byn^{Gal4} > ci^{Rep}$<br>$byn^{Gal4} > ci^{Rep}$ in $ap^{\Delta LSE}$	*y w* ; ap^{MS2} / ap^{DG3} ; byn^{Gal4} / +<br>*y w* ; byn^{Gal4} *UAS*-ci^{Rep} / +<br>*y w* ; ap^{MS2} / ap^{DG3} ; byn^{Gal4} *UAS*-ci^{Rep} / +	
**S10C-D**	*ctrl*<br>$ap^{\Delta LSE}$	*y w*<br>*y w* ; ap^{MS2} / ap^{DG3}	
**S11A-B**	ap^{md544} / ap^{DG3} (- *UAS-ap*)<br>ap^{md544} / ap^{DG3} (+ *UAS-ap*)	*y w* ; ap^{md544} *tub*-$Gal80^{ts}$ / ap^{DG3} *P{UAS-CD8-GFP}*<br>*y w* ; ap^{md544} *tub*-$Gal80^{ts}$ / ap^{DG3} *UAS-ap P{UAS-CD8-GFP}*	10<br>9
**S12A-B**	ap^{Gal4} > *UAS-CD8::GFP*<br>$ap^{\Delta LSE\text{-}Gal4\_2}$ > *UAS-CD8::GFP*<br>$ap^{LSE\text{-}Gal4}$ > *UAS-CD8::GFP*<br>byn^{Gal4} > *UAS-CD8::GFP*	*y w* ; $ap^{c1.4b\text{-}Gal4}$ / ap^{DG3} *UAS-CD8::GFP*<br>*y w* ; $ap^{MS3\text{-}Gal4}$ / ap^{DG3} *UAS-CD8::GFP*<br>*y w* ; $ap^{R1+LSE\text{-}Gal4}$ / ap^{DG3} *UAS-CD8::GFP*<br>*y w* ; + / ap^{DG3} *UAS-CD8::GFP* ; byn^{Gal4} / +	
**S12C**	ap^{Gal4}<br>$ap^{\Delta LSE\text{-}Gal4\_2}$<br>$ap^{LSE\text{-}Gal4}$	*y w* ; $ap^{c1.4b\text{-}Gal4}$ / ap^{DG3} *UAS-CD8::GFP*<br>*y w* ; $ap^{MS3\text{-}Gal4}$ / ap^{DG3} *UAS-CD8::GFP*<br>*y w* ; $ap^{R1+LSE\text{-}Gal4}$ / ap^{DG3} *UAS-CD8::GFP*	5 (25-68 flies each)<br>6 (24-67 flies each)<br>5 (37-66 flies each)
**S12D**	ap^{Gal4}<br>$ap^{\Delta LSE\text{-}Gal4\_2}$	*y w* ; $ap^{c1.4b\text{-}Gal4}$ / ap^{DG3} *UAS-CD8::GFP*<br>*y w* ; $ap^{MS3\text{-}Gal4}$ / ap^{DG3} *UAS-CD8::GFP*	6 (13-17 flies each)<br>6 (11-16 flies each)
**S12E**	ap^{Gal4}<br>$ap^{\Delta LSE\text{-}Gal4\_2}$	*y w* ; $ap^{c1.4b\text{-}Gal4}$ / ap^{DG3} *UAS-CD8::GFP*<br>*y w* ; $ap^{MS3\text{-}Gal4}$ / ap^{DG3} *UAS-CD8::GFP*	6 (15 flies each)<br>6 (15 flies each)
**S12F-H**	ap^{Gal4}<br>$ap^{\Delta LSE\text{-}Gal4\_2}$	*y w* ; $ap^{c1.4b\text{-}Gal4}$ / ap^{DG3} *UAS-CD8::GFP*<br>*y w* ; $ap^{MS3\text{-}Gal4}$ / ap^{DG3} *UAS-CD8::GFP*	10<br>10
**S13A**	$ap^{md544\text{-}Gal4}$ (- *UAS-ap*)<br>$ap^{md544\text{-}Gal4}$ (+ *UAS-ap*)<br>ap^{Gal4} (- *UAS-ap*)<br>ap^{Gal4} (+ *UAS-ap*)<br>$ap^{\Delta LSE\text{-}Gal4\_2}$ (- *UAS-ap*)<br>$ap^{\Delta LSE\text{-}Gal4\_2}$ (+ *UAS-ap*)<br>$ap^{LSE\text{-}Gal4}$ (- *UAS-ap*)<br>$ap^{LSE\text{-}Gal4}$ (+ *UAS-ap*)<br>$ap^{\Delta LSE\text{-}Gal4}$ (- *UAS-ap*)<br>$ap^{\Delta LSE\text{-}Gal4}$ (+ *UAS-ap*)	*y w* ; ap^{md544} / ap^{DG3} *UAS-CD8::GFP*<br>*y w* ; ap^{md544} / ap^{DG3} *UAS-ap UAS-CD8::GFP*<br>*y w* ; $ap^{c1.4b\text{-}Gal4}$ / ap^{DG3} *UAS-CD8::GFP*<br>*y w* ; $ap^{c1.4b\text{-}Gal4}$ / ap^{DG3} *UAS-ap UAS-CD8::GFP*<br>*y w* ; $ap^{MS3\text{-}Gal4}$ / ap^{DG3} *UAS-CD8::GFP*<br>*y w* ; $ap^{MS3\text{-}Gal4}$ / ap^{DG3} *UAS-ap UAS-CD8::GFP*<br>*y w* ; $ap^{R1+LSE\text{-}Gal4}$ / ap^{DG3} *UAS-CD8::GFP*<br>*y w* ; $ap^{R1+LSE\text{-}Gal4}$ / ap^{DG3} *UAS-ap UAS-CD8::GFP*<br>*y w* ; $ap^{DG1\text{-}Gal4}$ / ap^{DG3} *UAS-CD8::GFP*<br>*y w* ; $ap^{DG1\text{-}Gal4}$ / ap^{DG3} *UAS-ap UAS-CD8::GFP*	4 (25-32 flies each)<br>6 (10-46 flies each)<br>5 (32-90 flies each)<br>5 (25-68 flies each)<br>4 (32-55 flies each)<br>6 (24-67 flies each)<br>6 (27-66 flies each)<br>5 (37-66 flies each)<br>4 (17-45 flies each)<br>6 (18-30 flies each)
**S13B**	$ap^{md544\text{-}Gal4}$/ ap^{DG3} *UAS-ap*<br>$ap^{md544\text{-}Gal4}$/ ap^{DG3}	*y w* ; ap^{md544} / ap^{DG3} *UAS-ap UAS-CD8::GFP*<br>*y w* ; ap^{md544} / ap^{DG3} *UAS-CD8::GFP*	
**S13C**	ap^{Gal4} / ap^{DG3}	*y w* ; $ap^{c1.4b\text{-}Gal4}$ / ap^{DG3} *UAS-CD8::GFP*	
**S13D**	$ap^{\Delta LSE\text{-}Gal4\_2}$ / ap^{DG3}	*y w* ; $ap^{MS3\text{-}Gal4}$ / ap^{DG3} *UAS-CD8::GFP*	
**S13E**	$ap^{minLSE\text{-}Gal4}$ / ap^{DG3}	*y w* ; $ap^{R1+LSE\text{-}Gal4}$ / ap^{DG3} *UAS-CD8::GFP*	
**S13F**	$ap^{\Delta LSE\text{-}Gal44}$	*y w* ; $ap^{DG1\text{-}Gal4}$ / ap^{DG3} *UAS-CD8::GFP*	
**S14A-B**	ap^{Gal4}<br>$ap^{\Delta LSE\text{-}Gal4\_2}$	*y w* ; $ap^{c1.4b\text{-}Gal4}$ / ap^{DG3} *UAS-CD8::GFP*<br>*y w* ; $ap^{MS3\text{-}Gal4}$ / ap^{DG3} *UAS-CD8::GFP*	10<br>10
**S15A**	*ctrl*<br>*ctrl* (+250 mM NaCl)<br>$byn^{Gal4} > ci^{Rep}$ (+250 mM NaCl)	*y w*<br>*y w*<br>*y w* ; byn^{Gal4} *UAS*-ci^{Rep} / +	3 (25 flies each)<br>3 (24-25 flies each)<br>3 (25 flies each)
**S15C**		*y w* ; byn^{Gal4} *tubGal80*^{ts} / *UAS*-ci^{Rep}	3-5 (Table S4)
**S16A-B**	*ctrl*<br>$byn^{Gal4} > ci^{Rep}$<br>$byn^{Gal4} > ci^{Rep}$ in $ap^{\Delta LSE}$	*y w*<br>*y w* ; byn^{Gal4} *UAS*-ci^{Rep}/ +<br>*y w* ; ap^{MS2} / ap^{DG3} ; byn^{Gal4} *UAS*-ci^{Rep}/ +	8<br>8<br>8
**S17A-C**	*ctrl*<br>byn^{Gal4} / +<br>ci^{Rep} / +	*y w*<br>*y w* ; byn^{Gal4} / +<br>*y w* ; *UAS*-ci^{Rep} / +	10<br>10<br>10
**S18A-B**	*ctrl*<br>byn^{Gal4} / +<br>ci^{Rep} / +	*y w*<br>*y w* ; byn^{Gal4} / +<br>*y w* ; + ;*UAS*-ci^{Rep} / +	10<br>10<br>10
**S19**	*ctrl*<br>*anus-glued*	*y w*<br>*y w*	8<br>8

Table S3. Temperature shift experiment with *ap*^{md544} *P{tub-Gal80*^{ts}*} / ap*^{DG3} *P{UAS-ap)*

temperature regime	@18°C* const.	day 5 shift	day 6 shift	day 7 shift	day 8 shift	day 9 shift	day 10 shift	day 11 shift	day 12 shift	day 13 shift	day 14 shift	day 15 shift	@29°C* const.
age[1]		96-120	120-144	144-168	168-192	192-216	216-240	240-264	264-288	288-312	312-336	336-360	
survival[2]	8/265	10/10	12/12	12/12	20/20	13/14	17/18	16/16	14/20	1/19	1/18	0/14	409/414
fertility[3]	sterile	fertile	fertile	fertile	fertile	fertile	fertile	fertile	fertile	sterile	sterile	sterile	fertile
wings[4]	strong	normal	normal	strong	strong	strong	strong	strong	strong	strong	strong	strong	normal

* Observations made for cultures grown constantly at 18°C or 29°C show that genotype *ap*^{md544} *P{tub-Gal80*^{ts}*} / ap*^{DG3} *P{UAS-ap)* is appropriate for our experiment. At 18°C, Gal80^{ts} is functional and inhibits activation of *UAS-ap* by Gal4. Hence, no Ap protein is produced and the typical ap phenotypes observed for genotype *ap*^{md544}*/ap*^{DG3} flies are fully penetrant. In contrast, at 29°C, Gal4 produced by *ap*^{md544} can activate *UAS-ap* and all ap phenotypes are rescued.

[1] Indicates the age of the animals at the time they were shifted from 18°C to 29°C. For example, "day 5 shift" means that embryos were collected at 18°C for 24 h. Then, "day 5 shift" animals were aged at 18°C for 96 h before they were shifted to 29°C. Thus, at the time of the temperature shift, "day 5 shift" animals were 96-120 h old.

[2] Indicates the number of adult flies that survived >3 days relative to the number of flies present on day 0.

[3] Adult flies were also checked for their fertility. "sterile" means that no eggs were laid. "fertile" indicates that larval progeny were abundant. Note that precocious adult death and sterility phenotypes correlate (see also Wilson, 1981).

[4] *ap*^{md544}*/ap*^{DG3} flies display a wing phenotype close to that of *ap*^{null} flies. "strong" indicates that flies developed such wings. "normal" indicates that flies had wings like wild-type flies. Note that "day 6 shift" flies still develop normal wings. When using temperature sensitive allele *ap*^{ts78j}, Wilson reported that the temperature sensitive period for wing development extended "from late second through early third instar" (Wilson, 1981). It thus appears as if our assay system depending on Gal4/Gal80ts/UAS-ap is slightly delayed.

Table S4. Temperature shift experiment with *byn*^{Gal4} *P{tub-Gal80*^{ts}*} / P{UAS-ci*^{Rep}*)*

temperature regime	@18°C* const.	day 3 shift	day 5 shift	day 7 shift	day 9 shift	day 11 shift	day 12 shift	day 13 shift	@29°C* const.
age[1]		48-72	96-120	144-168	192-216	240-264	264-288	288-312	
survival[2]	>95%	>95%	>95%	>95%	>95%	>95%	>95%	>95%	>95%
papillae / ampulla[3]									
no papillae	-	2/5	2/4	2/4	3/4	1/4	-	-	4/4
1 tiny papilla	-	3/5	2/4	2/4	1/4	3/4	-	-	-
3 papillae	-	-	-	-	-	-	1/5	1/3	-
4 papillae	3/3	-	-	-	-	-	4/5	2/3	-
fertility[4]	fertile	fertile	fertile	fertile	fertile	fertile	fertile	fertile	fertile

* Observations made for cultures grown constantly at 18°C or 29°C show that genotype *by*^{Gal4} *P{tub-Gal80*^{ts}*} / P{UAS-ci*^{Rep}*)* is appropriate for our experiment. At 18°C, Gal80^{ts} is functional and inhibits activation of *UAS-ci*^{Rep} by Gal4. Hence, no ci^{Rep} protein is produced. Ampullae develop normally and contain 4 papillae. In contrast, at 29°C, Gal4 produced by *byn*^{Gal4} can activate *UAS-ci*^{Rep} and papilla-less ampullae are formed.

[1] Indicates the age of the animals at the time they were shifted from 18°C to 29°C. For example, “day 3 shift” means that embryos were collected at 18°C for 24 h. Then, “day 3 shift” animals were aged at 18°C for 48 h before they were shifted to 29°C. Thus, at the time of the temperature shift, “day 3 shift” animals were 48-72 h old.

[2] Since essentially all flies were surviving well, no quantitative analysis of survival was done. >95% indicates that survival is basically normal.

[3] Hindguts of a small number of female flies per shift were dissected and immediately analyzed and documented by light-microscopy. The number of papillae per ampulla is indicated. An example of a “tiny” papilla is shown in Fig. S15D.

[4] Adult flies were also checked for their fertility. “fertile” indicates that larval progeny were abundant.

Movie S1. 3D animation of ap^{Gal4} > G-TRACE embryo. 3D animation of fluorescence imaging stacks of an ap^{Gal4} > G-TRACE embryo reveals that *ap* is expressed in two stripes within the posterior part of the embryonic hindgut. Blue: DAPI, red: current expression of *ap*. Scale bar: 50 µm.

Movie S2. High-resolution recording of a *ctrl* fly. Minutes 50-55 of the 1 h recording are shown.

Movie S3. High-resolution recording of an $ap^{\Delta LSE}$ fly. Minutes 50-55 of the 1 h recording are shown.

Movie S4. High-resolution recording of a ap^{minLSE} fly. Minutes 50-55 of the 1 h recording are shown.
